# Determination of Fluorinated Pesticide Residues and Their Selected Transformation Products in High-Water-Content Foods Using Complementary LC–MS/MS and GC–MS/MS Methods

**DOI:** 10.3390/foods15173050

**Published:** 2026-08-28

**Authors:** Iwona Wenio, Damian Kwiatkowski, Daria Martyna Dawidziak, Dorota Derewiaka, Ewa Majewska, Iwona Bartosiewicz

**Affiliations:** 1 Department of Technology and Food Assessment, Faculty of Food Technology, Institute of Food Sciences, Warsaw University of Life Sciences, 02-787 Warsaw, Poland; dorota_derewiaka@sggw.edu.pl (D.D.); ewa_majewska1@sggw.edu.pl (E.M.); 2 Voivodeship Sanitary and Epidemiological Station, 00-875 Warsaw, Poland; damian.kwiatkowski@sanepid.gov.pl (D.K.); daria.dawidziak@sanepid.gov.pl (D.M.D.); iwona.bartosiewicz@sanepid.gov.pl (I.B.)

**Keywords:** fluorinated pesticide, degradation, transformation products (TPs), high-water-content foods

## Abstract

Fluorinated pesticides are an emerging class of environmental contaminants owing to their widespread agricultural use, high persistence, and ability to generate highly stable per- and polyfluoroalkyl substances (PFAS) during environmental degradation. Active ingredients containing fluorinated moieties undergo photolytic, hydrolytic, and microbial transformation, producing persistent metabolites such as trifluoroacetic acid (TFA), TFNA, TFNG, AMTT, and FM-6-1, which are highly mobile substances and may enter the food chain. To address this challenge, complementary LC–MS/MS and GC–MS/MS methods combined with QuEChERS and methanol extraction, and a modified QuPPe-based procedure for TFA and DFA were developed and validated for the determination of 76 fluorinated analytes, including fluorinated pesticides and selected transformation products with diverse physicochemical properties. Validation of the analytical workflow included the assessment of limits of quantification (LOQs), recovery, precision, expanded measurement uncertainty, and matrix effects at three spiking levels. LOQs ranged from 0.002 to 0.050 mg kg^−1^. Recoveries were 63.9–114.7% for GC–MS/MS and 40.3–109% for LC–MS/MS. The validated analytical workflow is suitable for monitoring fluorinated pesticides and selected transformation products in potatoes, onions, pears, watermelons, carrots, and cauliflower and may support routine food-safety control.

## 1. Introduction

Fluorinated active substances used in plant protection products are emerging as one of the most critical and insufficiently investigated contributors to contemporary environmental risk. Unlike traditional sources of fluoroorganic compounds, fluorinated pesticides are applied directly to soil and crops, resulting in immediate contact with the food sources. Their widespread use and long environmental half-lives—with some compounds persisting over 365 days in soil—highlight the urgency of addressing their fate and impacts [1]. Their chemical character—often containing CF_3_ groups or other strongly bound fluorine atoms—exhibits exceptional stability due to the high dissociation energy of the carbon–fluorine bond (~485 kJ/mol), its short bond length (≈1.35 Å), and strong dipolar character [2]. These physicochemical properties impose high activation barriers for C–F bond cleavage, restricting the degradation of fluorinated compounds to a limited set of pathways: reductive, hydrolytic, and oxidative defluorination. Each of these mechanisms leads to the formation of simple, highly persistent fluoroorganic acids—such as trifluoroacetic acid (TFA), difluoroacetic acid (DFA), and trifluoropropanoic acid (TFPrA)—which represent the terminal degradation products of fluorinated pesticides [3,4].

Fluorinated pesticides comprise a diverse class of plant protection products containing fluorinated functional groups that enhance biological activity and physicochemical properties. Among them, an increasing number of active substances fulfil the current OECD structural definition of per- and polyfluoroalkyl substances (PFAS) and are therefore classified as PFAS pesticides. These compounds typically contain fully fluorinated moieties, such as trifluoromethyl (–CF_3_) or difluoromethylene (–CF_2_–) groups. Increasing evidence indicates that the environmental transformation of selected PFAS pesticides may contribute to the formation of trifluoroacetic acid (TFA), an ultrashort-chain PFAS characterized by exceptional persistence and high mobility, highlighting the need for comprehensive analytical methods capable of determining both parent compounds and their transformation products [5].

Laboratory and field studies consistently demonstrate that fluorinated pesticides degrade only slowly under environmental conditions and follow distinct breakdown pathways compared with most organic compounds. Most of them degrade into highly mobile fluoroorganic acids such as trifluoroacetic acid (TFA) and difluoroacetic acid (DFA). These acids resist further breakdown, migrate readily through soil and water, and their presence in food raw materials is a direct consequence of the use of fluorinated active substances as plant protection products. However, other anthropogenic sources have also been identified [6]. In this context, short-chain PFAS do not represent an independent source of contamination; they can arise as terminal degradation products of fluorinated pesticides and, in some cases, from formulation components containing fluorinated additives. The environmental fate of these degradation products is particularly concerning. TFA, DFA, and related fluoroorganic acids are readily taken up by plants and accumulate in leaves and other aerial tissues through the transpiration stream [7]. Moreover, TFA formed from fluorinated pesticide ingredients is efficiently taken up and accumulated by vascular plants, confirming that these terminal degradation products enter multiple plant compartments rather than remaining confined to soil or water. Transfer from soil to edible crops has been confirmed for carrots, potatoes, and cucumbers, with the highest accumulation observed in vegetative tissues [8].

Several widely used pesticides, including isoxaflutole, fluopyram, penoxsulam, picolinafen, flurochloridone, flutianil, and flufenacet, have been identified as important –CF_3_-containing precursors of trifluoroacetic acid (TFA). Through photolytic and oxidative degradation, these compounds generate TFA as a terminal and environmentally persistent transformation product. Isoxaflutole alone is estimated to produce up to 364 t yr^−1^ of TFA in Europe. Penoxsulam, owing to its benzylic –CF_3_ moiety, exhibits particularly high TFA formation potential and releases fluoride (up to 53%) during photolysis. Picolinafen is one of the 18 pesticides responsible for the majority of TFA emissions in the European Union [9,10]. The transformation efficiency is equally alarming. Although the average TFA conversion factor is estimated at 30% per CF_3_ group, flufenacet reaches 82% molar conversion to TFA in aerobic soils. As a result, pesticide-derived TFA emissions are estimated at 970–3200 t yr^−1^ in Europe—with Poland and Germany contributing 17% and 16%, respectively—and 1500–5000 t yr^−1^ in the United States, where trifluralin (1537 t yr^−1^) and fomesafen (811 t yr^−1^) are the dominant sources [3].

Molecular structures of per- and polyfluoroalkyl substances (PFAS) commonly incorporate fluoro, trifluoromethyl (–CF_3_) and difluoromethyl (–CF_2_–) substituents, significantly reducing susceptibility to abiotic and biotic degradation, simultaneously promoting the formation of persistent fluorinated residues. In environmental systems, fluorinated pesticides undergo three principal transformation pathways: photolytic and oxidative degradation, hydrolytic cleavage, and slow microbial transformation. Each pathway generates characteristic stable fluorinated transformation products (TPs) that frequently exhibit greater mobility and environmental persistence than the parent compounds [11].

Photolytic and oxidative reactions constitute the dominant transformation pathways for pesticides containing –CF_3_ groups. Hydrolysis constitutes the second major transformation pathway, especially relevant for pesticides containing amide, sulfonylurea, or heterocyclic functional groups. Hydrolytic reactions generate more polar TPs that retain fluorinated fragments, thereby preserving their persistence and mobility. Flonicamid undergoes hydrolytic conversion to TFNA and TFNG, while triflumizole forms FM-6-1, tembotrione yields M5, and tritosulfuron produces AMTT. These hydrolysis products exhibit altered polarity, modified chromatographic retention, and distinct ionization behavior in LC-HRMS, complicating their detection in conventional analytical workflows. Hydrolysis is favored under elevated pH and moisture conditions, facilitating cleavage of amide, ester, and sulfonylurea linkages [12].

Microbial transformation represents the slowest pathway and occurs under both aerobic and anaerobic conditions. Soil and aquatic microorganisms can initiate hydroxylation, dehydrogenation, and partial defluorination, although the high bond dissociation energy of C–F bonds limits the extent of biotic degradation. Compounds such as fluazinam, flutolanil, and flurochloridone form fluorinated carboxylic acids with modified polarity, which are more mobile than the parent pesticides. Microbial TPs frequently retain –CF_3_ or –CF_2_– fragments, enabling their migration through soil profiles and into groundwater [13]. Collectively, these transformation pathways generate a range of persistent fluorinated products, including TFA, TFNA, TFNG, FM-6-1, AMTT, and M5. Their environmental occurrence underscores the need for advanced analytical methodologies capable of detecting both parent pesticides and their numerous TPs. High-resolution mass spectrometry workflows combining targeted, suspect, and non-target screening may provide complementary information on unidentified fluorinated transformation products in environmental matrices. However, only targeted MRM-based LC–MS/MS and GC–MS/MS methods were applied in the present study [14].

The data above demonstrate that fluorinated pesticides can generate persistent fluoroorganic residues that not only remain in the environment but also enter the food chain. Given the increasing use of fluorinated active substances, it is essential to implement complementary analytical methods capable of determining parent pesticides and their degradation products. Targeted methods such as LC-MS/MS and GC-MS/MS currently provide the most reliable first-line assessment of fluorinated pesticides and their simple degradation products. Their high sensitivity, selectivity, and MRM capability enable precise monitoring across a wide polarity range [15]. Targeted methods are limited to predefined analytes; therefore, high-resolution suspect and non-target screening may be considered in future studies to investigate additional unidentified fluorinated compounds [16]. Available evidence indicates that fluorinated pesticides represent a new generation of persistent fluoroorganic chemicals whose environmental and food-safety implications are far greater than previously recognized. Understanding their degradation pathways, mobility, and plant uptake is crucial for accurate risk assessment and for developing strategies to limit their presence in food and agricultural environments. Conventional QuEChERS-based multiresidue methods may not adequately cover highly polar transformation products such as TFA and DFA. The modified QuPPe-based procedure, using methanol containing 1% formic acid, was applied to the determination of TFA and DFA.

Fluorinated pesticides were selected as a chemically defined group because some may act as precursors of persistent and highly mobile fluorinated transformation products, including TFA. The substantial polarity differences between parent compounds and their transformation products present a specific analytical challenge and justify the use of complementary extraction and instrumental methods. Non-fluorinated pesticides were therefore outside the defined scope of this targeted study. The work aimed to address the limited ability of a single conventional multiresidue method to simultaneously cover fluorinated parent pesticides and selected highly polar transformation products. A complementary analytical workflow combining LC–MS/MS and GC–MS/MS with QuEChERS, methanol extraction, and a modified QuPPe-based procedure was developed and validated for analytes covering a broad polarity range. The relative performance of the extraction procedures was compared for different analyte groups, and the validated workflow was subsequently applied to commercial high-water-content food samples to determine the occurrence and concentrations of the target compounds.

## 2. Materials and Methods

### 2.1. Materials

Analytical standards of the investigated compounds used for calibration and spiking procedures were purchased from Dr. Ehrenstorfer (LGC Standards, Augsburg, Germany). All reference standards were of analytical grade with purity ranging from 96.0 to 99.9%. (Table S1) Acetonitrile (ACN) and methanol (MeOH) used for extraction procedures were of LC–MS grade with purity ≥99.9% and were purchased from J.T. Baker Chemicals (Phillipsburg, NJ, USA). Formic acid used for the QuPPe extraction procedure was obtained from Merck (Darmstadt, Germany) with a purity of ≥98%. Acetic acid used for QuEChERS extract modification and LC–MS/MS analysis was purchased from Merck (Darmstadt, Germany). Toluene, acetone, and n-decane used for GC–MS/MS analysis were of chromatographic grade and obtained from J.T. Baker Chemicals (Phillipsburg, NJ, USA). For the QuEChERS extraction procedure, anhydrous magnesium sulfate (MgSO_4_, purity ≥99.5%), sodium chloride (NaCl, purity ≥99.5%), trisodium citrate dihydrate, and disodium citrate sesquihydrate were purchased from Merck (Darmstadt, Germany). Primary secondary amine (PSA) and Octadecylsilane sorbents (C18) used for dispersive solid-phase extraction (dSPE) clean-up were obtained from Supelco (Bellefonte, PA, USA). For the method 2 extraction procedure, sodium hydroxide (NaOH, purity ≥99%) used for pH adjustment was purchased from Merck (Darmstadt, Germany). C18 sorbent used for dispersive clean-up was obtained from Supelco (Bellefonte, PA, USA). For the QuPPe extraction procedure, 1% (*v*/*v*) formic acid in methanol was prepared using LC–MS-grade methanol and formic acid. Deionized water used for the dilution of extracts before LC–MS/MS analysis was produced using a Milli-Q purification system (Millipore, Darmstadt, Germany). Extracts obtained from all extraction procedures were filtered before instrumental analysis using 0.20 µm PTFE syringe filters and 0.22 µm mixed cellulose ester (MCE) syringe filters purchased from Merck Millipore (Darmstadt, Germany). All solvents and reagents used were of analytical, chromatographic, or LC–MS grade.

### 2.2. Sample Collection

The experimental material comprised various fruit and vegetable commodities characterized by high-water content according to SANTE/11312/2021 v2026 [17] in Annex A, collected from diverse geographical regions across Poland. The fruit and vegetable samples originated from the European monitoring programme and represented the following commodities. A total of 132 samples were analysed, including pears (*n* = 31), potatoes (*n* = 31), carrots (*n* = 23), onions (*n* = 19), cauliflowers (*n* = 24), and watermelons (*n* = 4). The sampling campaign was conducted continuously from January to the end of June 2026. The number of samples within individual commodity groups reflected the actual sampling structure of the monitoring programme and the seasonal availability of the respective commodities during the study period. Consequently, the smaller number of watermelon samples (*n* = 4) resulted from their limited seasonal availability within the January–June sampling period. Upon arrival at the laboratory, each laboratory sample was thoroughly homogenized, and the resulting analytical portions were stored at −20 °C until extraction. Each routine sample was subjected to a single independent extraction using the method appropriate for the target analytes, and each final extract was analysed once by the corresponding instrumental method. In contrast, method validation was performed at three spiking levels, with five independently prepared and analysed replicates at each level.

### 2.3. Instrument and Experimental Conditions

#### 2.3.1. Method 1 Based on QuEChERS LC-MS/MS

For the LC-MS/MS analyses based on the QuEChERS method, an Agilent 1260 Infinity II, a G71128 binary pump degassing unit, and an autosampler G7129A, coupled to a Triple Quad QQQ Agilent 6470B, were employed (Agilent Technologies, Santa Clara, CA, USA). The separation was achieved using an Agilent InfinityLab Poroshell 120 EC C18 column (100 mm × 2.1 mm ID × 1.8 μm dp) (Agilent Technologies, Santa Clara, CA, USA) at a temperature of 50 °C in column compartment G7116A (Agilent Technologies, Santa Clara, CA, USA). At a flow rate of 0.5 mL min^−1^, the mobile phase was composed of water with ammonium formate (5 mM) and 0.01% formic acid (phase A) and ammonium formate (5 mM) and 0.01% formic acid in a mixture of acetonitrile: water (95:5 *v*/*v*) (phase B). The gradient program was as follows: 6% B from 0.0 to 0.5 min; a linear increase from 6% to 95% B between 0.5 and 25.0 min; 95% B from 25.0 to 27.0 min; and a linear decrease from 95% to 6% B between 27.0 and 32.0 min. The injection volume was 2 μL. The determination of the target analytes was performed in MRM acquisition mode using an electrospray ionization source operating in both positive (+) and negative (−) ionization modes. The MS parameters were as follows: gas temperature 350 °C, sheath gas temperature 350 °C, nebulizing gas flow nitrogen (N_2_) 9 L min^−1^, sheath gas flow (N2) 11 L min^−1^, capillary voltage: positive 4000 V and negative 3500 V. Nozzle voltage: 500 V in negative-ion mode. Two different MRM transitions (quantifier and qualifier) were optimized and monitored for each compound (Table S2). System control and data handling were performed using the MassHunter 12 software.

#### 2.3.2. Method 1 Based on QuEChERS GC-MS/MS

For the GC-QqQMS analyses based on the QuEChERS method, an Agilent 8890 GC system (Agilent Technologies, Santa Clara, CA, USA), equipped with an Agilent 7693A automatic liquid sampler (ALS) and coupled with an Agilent 7000D triple quadrupole mass spectrometer equipped with an electron ionization source (70 eV, 300 °C), was used. Sample extracts were injected using an Agilent 7693A automatic liquid sampler (Agilent Technologies, Santa Clara, CA, USA), with a 10 μL syringe, into a front multi-mode inlet (MMI). The injector temperature was set at 280 °C. A 1 µL aliquot of the sample extract was injected in splitless mode at the injector, with a splitless time of 0.75 min. The purge flow to the split vent was 30 mL/min, while the septum purge flow was maintained at 3 mL/min. Constant-pressure mode injection was applied with an inlet pressure of 31.3 psi. Separation was performed on an HP-5MSUI (Agilent Technologies) fused silica capillary column of 30 m × 0.25 mm, at 0.25 µm of the film thickness (Column 1), connected in series via an Aux EPC 4 module to a deactivated fused silica capillary restrictor column of 0.7 m × 0.15 mm (Column 2). The restrictor column was used to enable controlled post-run backflush and to facilitate the removal of high-boiling matrix components from the analytical column. Helium was used as the carrier gas at a constant pressure, providing an initial flow rate of 3.3 mL/min for Column 1 and 3.2 mL/min for Column 2. The column oven temperature program was as follows: the initial temperature was set at 70 °C and maintained for 2 min; then, it was increased to 150 °C at a rate of 25 °C/min, increased to 200 °C at a rate of 3 °C/min, and finally increased to 280 °C at a rate of 8 °C/min and held for 11 min. The total program run time was 42.87 min. Immediately following the analytical run, a post-column backflush procedure was executed for 5 min at an oven and restrictor temperature of 280 °C. During this post-run phase, the inlet pressure was decreased to 2 psi while the auxiliary pressure (Aux EPC 4) was increased to 50 psi, generating a reverse column flow of −3.171 mL/min to remove safety-boiling matrix components. The mass spectrometer was operated with an electron impact (EI) source using the dynamic multiple reaction monitoring (dMRM) mode with a gain factor of 45 (calculated EMV of 2014.7 V). In Table S3, a characterization of the ions used for qualitative analysis of the pesticides is presented. The ion source and transfer line temperatures were set at 300 °C and 280 °C, respectively, while both quadrupoles (MS1 and MS2) were maintained at 180 °C. The software Agilent MassHunter (version B.09.00 or equivalent) was used for instrument control, data acquisition and processing.

#### 2.3.3. Method 2—LC-MS/MS

For the LC-MS/MS analyses, an Agilent 1260 Infinity II, a G71128 binary pump degassing unit, and an autosampler G7129A, coupled to a Triple Quad QQQ Agilent 6470B, were employed. The separation was achieved using an Agilent Zorbax Eclipse Plus C18 column (150 mm × 2.1 mm ID × 1.8 μm dp) (Agilent Technologies, Santa Clara, CA, USA) at a temperature of 55 °C in a G 7116A column compartment. At a flow rate of 0.2 mL min^−1^, the mobile phase was composed of water with ammonium formate (5 mM) and 0.01% formic acid (phase A) and methanol (phase B). The gradient program was as follows: 0.2 min hold at 5% phase B, 5–90% phase B gradient over 0.1–16 min, 8 min hold at 90% phase B, 90–5% phase B gradient over 24.1–45 min. The injection volume was 2 μL. The determination of the target analytes was performed in MRM acquisition mode using an electrospray ionization source operating in both negative (−) and positive (+) ionization modes. The MS parameters were as follows: gas temperature 250 °C, sheath gas temperature 350 °C, nebulizing gas flow nitrogen (N_2_) 6 L min^−1^, sheath gas flow nitrogen (N_2_) 11 L min^−1^, capillary voltage positive 3000 V and negative 3500 V. Two different MRM transitions (quantifier and qualifier) were optimized and monitored for each compound (Table S4). System control and data handling were performed using the MassHunter 12 software.

#### 2.3.4. Method 3 Based on QuPPe LC-MS/MS

For the LC-MS/MS analyses based on the QuPPe method, an Agilent 1260 Infinity, a G1312B binary pump degassing unit, and an autosampler G1367E, coupled to a Triple Quad QQQ Agilent G6460C, were employed. The separation was achieved on a ThermoScientific Hypercarb column (100 mm × 2.1 mm ID × 5 μm dp) (Thermo Fisher Scientific, Waltham, MA, USA) at a temperature of 50 °C in column compartment G7116A. At a flow rate of 0.2 mL min^−1^, the mobile phase was composed of a mixture of water: methanol 95:5 (*v*/*v*) with 1% formic acid (phase A) and 1% formic acid in methanol (phase B). The gradient program was as follows: 0% B held for 0.1 min at flow rate of 0.20 mL min^−1^, then change 0–30% B gradient over 0.1–10 min, in 11 min change flow rate to 0.40 mL min^−1^ and hold 7 min at in 30% B to the 18 min, then change the gradient to the from 30% to 90% phase B% on 19 min and held at 90%B for 3 min to 22 min, then in 22.1–30.0 min, 0% B; and on 30.0 min, 0% B change flow rate 0.20 mL min^−1^. The injection volume was 25 μL. The determination of the target analytes was performed in MRM acquisition mode using an electrospray ionization source operating in both positive (+) and negative (−) ionization modes. The MS parameters were as follows: gas temperature 350 °C, sheath gas temperature 350 °C, nebulizing gas flow (N2) 10 L min^−1^, sheath gas flow nitrogen (N_2_) 12 L min^−1^, capillary voltage: positive 3000 V and negative 2000 V. Nozzle voltage (v) 300 for the negative setpoint. Two different MRM transitions (quantifier and qualifier) were optimized and monitored for each compound and their ratio (Table S5). System control and data handling were performed using the MassHunter 12 software.

### 2.4. Preparation of the Sample

#### 2.4.1. Extraction Procedure Based on the QuEChERS—Method 1

Sample preparation was carried out according to a modified QuEChERS-type extraction protocol. Approximately 10.0 ± 0.1 g of homogenized sample material was weighed into a 50 mL polypropylene centrifuge tube. For spiking samples, the appropriate volume of spiking solution MIX1 LC and MIX 1 GC (Table S1) was added (I level (LOQ) 10 µL, II level (10·LOQ) 100 µL, III level (40·LOQ) 400 µL). Subsequently, 10.0 mL of acetonitrile was added to the tube, and the mixture was vigorously shaken for 2 min to facilitate efficient extraction of the target analytes. A salt mixture consisting of 4.0 ± 0.2 g of anhydrous MgSO_4_, 1.0 ± 0.05 g of NaCl, 1.0 ± 0.05 g of trisodium citrate dihydrate, and 0.50 ± 0.03 g of disodium citrate sesquihydrate was then added to the tube, which was immediately cooled in an ice–water bath. The mixture was vigorously shaken for 2 min, then centrifuged for 5 min at 3000 rpm to achieve complete separation of the organic and aqueous phases. An aliquot of the upper acetonitrile phase was subjected to dispersive clean-up using PSA or C18 sorbent (25 mg) and MgSO_4_ (150 mg per 1 mL of extract), shaken for 2 min, cooled, and centrifuged for 2 min at 3500 rpm. The clarified supernatant was filtered through a 0.2 µm PTFE syringe filter. For GC-MS/MS, 2 mL of extract was combined with acetonitrile containing 5% acetic acid, evaporated at 40 °C with one drop of n-decane added to the dry extract, and reconstituted in a 2 mL toluene: acetone (1:1, *v*/*v*) mixture. For LC-MS/MS, 10 µL of 5% acetic acid in acetonitrile was added per mL of extract before filtration and instrumental analysis.

#### 2.4.2. Sample Extraction Using Method 2

Homogenized sample material (5.00 ± 0.01 g) was weighed into a 50 mL polypropylene centrifuge tube. For spiking samples, the appropriate volume of spiking solution MIX2 LC (Table S1) was added (I level (LOQ) 10 µL, II level (10·LOQ) 100 µL, III level (40·LOQ) 400 µL). Spiking samples were mixed thoroughly and allowed to equilibrate for 30 min to ensure complete analyte–matrix interaction. 5 mL of methanol was added to the homogenate, and the suspension was briefly agitated. The pH of the mixture was measured and adjusted to 5.5–7.5 when necessary, using 5 M NaOH. Samples within the required pH range were sealed and subjected to vigorous shaking for 15 min, followed by 5 min centrifugation at 3500 rpm to obtain clear phase separation. An aliquot of the extract was transferred to a 15 mL centrifuge tube and purified by dispersive clean-up using C18 sorbent (25 mg per 1 mL of extract). After 2 min shaking, the mixture was centrifuged at 5500 rpm, and the supernatant was collected and filtered through a 0.22 µm PTFE syringe filter. The final extract was subjected to LC-MS/MS analysis.

#### 2.4.3. Extraction Sample Based on the QuPPe—Method 3

Homogenized sample material (10.0 ± 0.1 g) was weighed into a 50 mL polypropylene centrifuge tube. For spiking samples, the appropriate volume of spiking solution MIX3 LC (Table S1) was added (I level (LOQ) 40 µL, II level (10·LOQ) 400 µL, III level (40·LOQ) 1600 µL). The fortified samples were allowed to equilibrate for 30 min to ensure sufficient interaction between the analytes and the sample matrix. Subsequently, 10.0 mL of 1% (*v*/*v*) formic acid in methanol was added, and the samples were vigorously shaken for 2 min to facilitate analyte extraction. The extracts were centrifuged for 5 min at 3000 rpm. The supernatant was filtered through a 0.22 µm mixed cellulose ester (MCE) syringe filter into a polypropylene tube. For LC–MS/MS analysis, an aliquot of the filtered extract was diluted with deionized water by transferring either 100 µL of extract into 900 µL of water in a glass autosampler vial. The prepared extracts were subsequently analyzed by LC–MS/MS.

#### 2.4.4. Method Validation, Quality Assurance

Method validation was performed in accordance with the SANTE/11312/2021 v2026 guidance document. The evaluated performance characteristics included selectivity, linearity, limits of quantification (LOQs), recovery, precision, matrix effects and measurement uncertainty. Matrix effects were assessed by comparing the slopes of solvent-based and matrix-matched calibration curves. Recovery, precision and measurement uncertainty were evaluated using independently prepared matrix-fortified samples at three concentration levels (1 × LOQ, 10 × LOQ and 40 × LOQ), each analysed in five replicates. Measurement uncertainty was estimated in accordance with the recommendations of the SANTE/11312/2021 v2026 guidance document. The obtained validation parameters fulfilled the acceptance criteria specified in the SANTE guidelines, confirming the suitability of the developed analytical methods for the determination of fluorinated pesticides and selected transformation products in high-water-content food matrices. The validation results are summarized in Table 1, Table 2, Table 3 and Table 4. To ensure the reliability of routine analyses, spiking quality control (QC) samples were included in every analytical sequence where the sequence contained more than *n* > 5 samples. The calibration was based on matrix matching. The recoveries obtained for all analysed matrices remained within the acceptance criteria established during method validation, with relative standard deviations (RSDs) not exceeding 20%, confirming the consistent analytical performance of the methods throughout the study. The matrix effect was evaluated in accordance with the SANTE guidelines by comparing the slopes of calibration curves prepared in the matrix and in solvent. The matrix effect (ME%) was calculated using the following equation: ME (in %) = [(*slope_matrix*/*slope_solvent*) − 1] × 100, where *slope_matrix* and *slope_solvent* represent the slopes of the calibration curves prepared in matrix extract and solvent, respectively. The obtained value was above the criterion of ±20%, indicating the presence of a significant matrix effect. A reagent blank and a matrix blank were analyzed to verify the absence of contamination and matrix interferences. No interfering signals were observed in either blank. At least one reagent blank was included in each analytical sequence and subjected to the complete sample-preparation procedure to monitor potential background contamination originating from reagents, solvents, filters, sample-preparation materials, and the analytical system. Particular attention was paid to TFA and DFA because of their high polarity and potential susceptibility to background contamination. Blank responses were evaluated before quantitative interpretation of the corresponding analytical sequence. In accordance with the SANTE specificity criterion, responses in reagent blanks and blank control samples at the retention time of the target analyte were required not to exceed 30% of the response corresponding to the reporting limit (RL). Potential contamination was minimized by using LC–MS-grade solvents, freshly prepared reagents, and pretested filters and sample-preparation materials. Blank injections following the highest calibration standard were also used to monitor instrumental carryover. Representative chromatograms for TFA and DFA obtained from the reagent blank, blank matrix, and the corresponding matrix-matched calibration standard at the validated LOQ level are provided in the Supplementary Materials (Figures S1 and S2). Quantification was performed using matrix-matched calibration curves consisting of six concentration levels (0.005, 0.010, 0.015, 0.10, 0.30, and 0.40 mg kg^−1^). Analyte-specific calibration levels were applied where required: TFA (0.025, 0.050, 0.075, 0.50, 1.5, and 2.0 mg kg^−1^), DFA (0.010, 0.020, 0.030, 0.20, 0.60, and 0.80 mg kg^−1^), fipronil (0.0010, 0.0020, 0.0030, 0.020, 0.060, and 0.080 mg kg^−1^), fipronil sulfone (0.0015, 0.0030, 0.0045, 0.030, 0.090, and 0.12 mg kg^−1^), and flutolanil (0.0025, 0.0050, 0.0075, 0.050, 0.15, and 0.20 mg kg^−1^). The calibration range included a sub-LOQ point at 0.025 mg kg^−1^, whereas the validated reporting LOQ was 0.050 mg kg^−1^; consequently, results below 0.050 mg kg^−1^ were not reported quantitatively. Calibration curves were constructed using an unweighted linear regression model and fulfilled the applicable acceptance criteria specified in SANTE/11312/2021 v2026. Analyte identification was based on a minimum of two MRM transitions, agreement of the retention time with the corresponding matrix-matched calibration standard (retention time deviation not exceeding ±0.1 min), and compliance with the relative ion-intensity acceptance criterion specified in Section D11 and Table 3 of SANTE/11312/2021 v2026, with the relative ion intensity not deviating by more than ±30% from the reference value. Carryover was evaluated by analysing reagent blanks after the highest calibration standard, and no significant carryover was observed. To verify the applicability of the validated methods to additional commodities within the high-water-content commodity group, a matrix-fortified quality-control sample at the LOQ level was prepared and analysed for each food matrix included in every analytical sequence. Quantification was performed using commodity-specific matrix-matched calibration prepared in the corresponding food matrix. These routine quality-control procedures confirmed the applicability and consistent analytical performance of the validated methods for pear, carrot, onion, cauliflower, and watermelon, in accordance with Section G2 and Appendix A of SANTE/11312/2021 v2026 [17]. Quantification was performed without isotopically labelled internal standards. According to the laboratory procedure, recovery correction would be applied if the recovery obtained for the corresponding fortified quality-control sample was below 80%. However, recoveries obtained for the fortified quality-control samples included in the routine analytical sequences were above 80%; therefore, the concentrations reported for the analysed commercial samples were not corrected for recovery. Measurement uncertainty was estimated using the empirical (“top-down”) approach recommended in Section E and Appendix C of SANTE/11312/2021 v2026, based on validation and routine quality-control data [17].

The combination of method validation, routine quality control using matrix-fortified samples, and successful participation in external proficiency testing confirmed the robustness of the developed analytical workflow and its suitability for the routine determination of fluorinated pesticides and selected transformation products in high-water-content food matrices. The developed methodology is currently applied in the routine monitoring of pesticide residues in food carried out by the official control programme in Poland.

## 3. Results

### 3.1. Analytical Performance and Comparison Methods

The direct comparison of compounds determined by Method 1, based on the QuEChERS extraction procedure, and Method 2, based on methanol extraction, revealed that substantial differences in recoveries were limited to only a few analytes. For most compounds determined by both methods, recoveries fulfilled the SANTE acceptance criteria, indicating comparable analytical performance despite the different extraction principles. The most pronounced improvement obtained by Method 2 was observed for the sulfonylurea herbicides. Flazasulfuron showed recoveries of 81.4, 71.9, and 80.9% following extraction with Method 1, whereas Method 2 increased recoveries to 93.7, 101.7, and 103% at the corresponding spiking levels LOQ, 10·LOQ and 40·LOQ. Similarly, penoxsulam recoveries increased from 79.5 to 86.3% to 93.2–101%, and prosulfuron recoveries improved from 82.2 to 89.7% to 96.3–96.9%. These results demonstrate that Method 2 provides considerably more efficient extraction of highly polar sulfonylurea herbicides than the acetonitrile-based QuEChERS procedure employed in Method 1. An even greater difference was observed for the ultrashort-chain fluorinated transformation products (TPs). TFNA and TFNG were successfully quantified with Method 2, yielding recoveries of 93.6–99.6% and 89.0–100.9%, respectively. In contrast, Method 1 proved better for several relatively hydrophobic fluorinated pesticides. The clearest example can be drawn for mefentrifluconazole, for which recoveries were 81.6–87.1% determined with the use of Method 1, and only 41.2–47.3% with Method 2. A similar reduction was observed for triflumizole metabolite FM-6-1, with recoveries ranging from 97.7 to 101% (Method 1) to 48.5–58.5% (Method 2). These compounds, therefore, remain better suited to extraction using Method 1. For the remaining compounds determined by both methods, including tritosulfuron, tetraniliprole, and fluopyram benzamide (M25), differences in recoveries were insignificant, and both methods fulfilled the validation criteria. Consequently, no clear analytical advantage of either extraction procedure could be demonstrated for these analytes. Method 1 was shown to be more suitable for relatively hydrophobic fluorinated pesticides, whereas Method 2 is essential for highly polar fluorinated herbicides and ultrashort-chain fluorinated transformation products. Therefore, a complementary application of both methods provides comprehensive analytical coverage of fluorinated pesticides with markedly different physicochemical properties. The cases described above illustrate the general procedure for selecting the quantitative method. For all analytes covered by both extraction procedures, routine quantitative determination was performed using the method providing the more satisfactory recovery, preferably within 70–120%, together with RSDr ≤20% at the validated fortification levels. In accordance with Section G6 of SANTE/11312/2021 v2026, recoveries outside 70–120% were considered acceptable only in justified exceptional cases when they were consistent RSDr ≤20%) and remained within the absolute recovery range of 30–140%. Results obtained using the alternative extraction procedure were not used for routine quantitative reporting but provided supplementary evidence supporting analyte identification in separately prepared extracts. Accordingly, Method 1 was selected for the quantitative determination of mefentrifluconazole and FM-6-1 because it provided recoveries of 81.6–87.1% and 97.7–101%, respectively. This method-selection principle was applied to all analytes included in both procedures.

Method 1 provided excellent recoveries for non-polar and moderately polar fluorinated pesticides, including pyrethroids, benzoylureas, strobilurins, diamides, and other hydrophobic compounds, which efficiently partition into the organic phase during salting-out extraction. This observation is consistent with previous studies demonstrating that QuEChERS is particularly suitable for pesticides with medium-to-high logP values and structurally complex hydrophobic analytes [18]. Although lipophilicity (logP) was an important determinant of extraction efficiency, it was not the only factor governing analyte behavior. The extraction performance was also influenced by the degree of ionization (pKa), hydrogen-bonding capacity, molecular polarity, topological polar surface area, and specific analyte–solvent interactions, which collectively determine solvent affinity and extraction efficiency [19,20].

The extraction efficiency of the investigated fluorinated pesticides appeared to be associated with their physicochemical properties, particularly lipophilicity, expressed as the octanol/water partition coefficient (logP) (Table S6). A distinct relationship between logP and analytical performance was observed, reflecting the fundamentally different extraction mechanisms of the procedures. The QuEChERS-based Method 1 exhibited superior performance for analytes of moderate to high lipophilicity. Most compounds with logP values above approximately 3.5, including chlorfluazuron (logP 6.7), fluazinam (6.0), hydramethylnon (6.4), lufenuron (6.4), triflumuron (5.6), flutolanil (3.7), and tau-fluvalinate (7.7), fulfilled the SANTE validation criteria at all fortification levels. The high recoveries obtained for these compounds can be attributed to their preferential partitioning into the acetonitrile-rich phase during salting-out extraction, which favours analytes exhibiting low aqueous solubility and pronounced hydrophobicity. These results confirm that the QuEChERS extraction procedure is particularly well suited for fluorinated pesticides possessing relatively high logP values. In contrast, Method 2, based on methanolic extraction, proved substantially more efficient for polar and moderately polar analytes. Sulfonylurea herbicides, including flazasulfuron (logP 2.0), penoxsulam (2.9), pyroxsulam (1.7) and tritosulfuron (3.1), showed markedly improved recoveries compared with Method 1. Likewise, the highly polar fluorinated transformation products TFNA and TFNG were successfully determined only using Method 2. Mefentrifluconazole, despite its intermediate lipophilicity (logP 3.4–4.0), exhibited a pronounced decrease in recovery from 81.6 to 87.1% with Method 1 to only 41.2–47.3% with Method 2. A similar trend was observed for the triflumizole metabolite FM-6-1 (logP 3.3), for which recoveries decreased from 97.7 to 101% to 48.5–58.5%. These observations suggest that extraction efficiency may also be influenced by additional molecular characteristics, including the presence of ionisable functional groups, hydrogen-bonding capacity, molecular conformation, and specific analyte–solvent interactions, which collectively influence solvent affinity and extraction thermodynamics.

The validated analytical workflow was successfully applied to the determination of fluorinated pesticides and their transformation products in commercial samples of pear, potato, carrot, onion, cauliflower and watermelon presented in Table 5. A total of 132 commercial food samples were analysed, including pears (*n* = 31), potatoes (*n* = 31), carrots (*n* = 23), onions (*n* = 19), cauliflowers (*n* = 24), and watermelons (*n* = 4). All reported concentrations were equal to or above the validated LOQs for the respective analytes. Quantification was performed using commodity-specific matrix-matched calibration. Recoveries obtained for fortified quality-control samples analysed during routine sequences were above 80%; therefore, the reported concentrations were not corrected for recovery. Expanded measurement uncertainty was estimated using an empirical top-down approach according to SANTE/11312/2021 v2026. Compliance with the applicable MRLs was assessed taking into account the expanded measurement uncertainty determined by the laboratory (U = 50%, k = 2), in accordance with the laboratory decision rule. Nine target analytes were detected, including five parent pesticides (fludioxonil, fluazinam, fluopyram, fluopicolide, and flupyradifurone) and four transformation products (TFNA, TFNG, DFA, and TFA). Their occurrence varied among the investigated food matrices; however, in the absence of crop-treatment records, these differences cannot be directly attributed to specific pesticide application patterns. Overall, fludioxonil was the most frequently detected analyte, occurring in 12 of the 132 analysed samples (9.1%), followed by TFA detected in nine samples (6.8%). Fluazinam and TFNA were each detected in seven samples (5.3%), DFA in five samples (3.8%), TFNG in four samples (3.0%), and fluopyram in three samples (2.3%). Fluopicolide and flupyradifurone were each detected in one sample (0.8%). Pear samples exhibited the highest diversity of detected compounds. In addition to fludioxonil, residues of fluazinam, flupyradifurone, fluopyram, TFNA, TFNG, and DFA were identified. Fludioxonil was the most frequently detected parent compound, with concentrations ranging from 0.013 to 0.220 mg kg^−1^. Flupyradifurone was detected in one pear sample at 0.041 mg kg^−1^. Fluopyram was detected in two potato samples at 0.016 and 0.022 mg kg^−1^ and in one pear sample at 0.082 mg kg^−1^. Transformation products occurred over concentration ranges of 0.014–0.140 mg kg^−1^ for TFNA, 0.019–0.100 mg kg^−1^ for TFNG, 0.022–0.350 mg kg^−1^ for DFA, and 0.072–0.190 mg kg^−1^ for TFA. Their occurrence in the investigated commodities is reported descriptively and should not be interpreted as direct evidence of the application or degradation of specific fluorinated pesticides, as crop-treatment records and precursor–product relationships were not available. Potato samples contained fluazinam together with TFNA, TFNG and TFA, whereas carrot samples contained fluazinam and TFA. Fluopicolide was detected in one potato sample at a concentration of 0.018 mg kg^−1^. In onion samples, only transformation products (TFNA, TFNG and TFA) were detected, while watermelon samples contained TFNA exclusively.

### 3.2. Proficiency Testing and Performance Assessment

The laboratory also participated in proficiency testing (PT) schemes organized by the European Union Reference Laboratory for Residues of Pesticides in Fruits and Vegetables (EUPT-FV/SRM). The proficiency test material consisted of homogenized tomato samples. Calculation of z-scores was performed according to the standard definition as (xi − xpt)/σpt, where xi is the reported result, xpt the assigned value, and σpt the standard deviation for proficiency assessment. Z-score classification was applied as follows: |z| ≤ 2.0, acceptable; 2.0 < |z| < 3.0, questionable; and |z| ≥ 3.0, unacceptable. The laboratory (participant No. 36) achieved satisfactory performance, with all reported z-scores falling within the acceptable range (|z| ≤ 2.0), demonstrating the accuracy, reliability, and comparability of Methods 1 and 2 for the determination of acrinathrin (−0.1), beflubutamid (−0.3), benfluralin (−0.1), diflufenican (0.0), flazasulfuron (−0.2), TFNA (0.7), TFNG (1.9), fluazifop (free acid) (0.6), fluometuron (−0.2), flupyradifurone (0.1), flutolanil (−0.1), fluxapyroxad (0.0), haloxyfop (free acid) (0.0), isopyrazam (−0.5), lambda-cyhalothrin (0.3), penthiopyrad (−0.6), and picoxystrobin (−0.1) in tomato samples.

The proficiency-test results were additionally evaluated with respect to false-positive and false-negative findings. No false-positive results were observed, i.e., no analytes absent from the test material were incorrectly reported as present. Likewise, no false-negative results were identified among the analytes included in the laboratory scope, indicating that the target analytes present in the proficiency-test material were successfully detected and reported.

## 4. Discussion

Potential false-positive and false-negative identifications were considered in light of the findings of Tóth et al. [21], who demonstrated that co-eluting matrix components may occasionally fulfil conventional retention-time and ion-ratio criteria and that two MRM transitions alone may not always provide sufficient selectivity. Although their examples concerned different pesticides, this analytical risk is relevant to broad-scope residue methods. In accordance with SANTE/11312/2021 v2026, analyte identification required two product ions (two MRM transitions), retention-time agreement within ±0.1 min, and ion ratios within ±30% (relative) of the reference value obtained from calibration standards analysed in the same sequence. Additional transitions were monitored whenever sufficient sensitivity was available, strengthening analyte confirmation and helping to distinguish target signals from matrix interferences. Mol et al., based on more than 135,000 chromatograms obtained for 120 pesticides in 21 fruit and vegetable matrices, supported the use of an absolute retention-time tolerance of ±0.1 min and demonstrated that reliable ion-ratio evaluation requires sufficient signal intensity for both product ions. Low qualifier-ion responses close to the detection limit may otherwise increase the risk of false-negative identification [22].

Matrix-related identification problems are also relevant to GC–MS/MS analysis. Uclés et al. [23] evaluated 25 blank food matrices using GC–QqQ–MS/MS and LC–QqQ–MS/MS and demonstrated that co-eluting matrix components producing the same monitored transitions may compromise unequivocal analyte identification. Such interferences were more frequently observed under GC–MS/MS conditions. In the present GC–MS/MS method, acrinathrin and cyhalothrin (sum of isomers) shared the nominal transition *m*/*z* 208→181, which would provide limited selectivity if used alone. However, their reference retention times were 30.77 and 30.44 min, respectively (ΔRT = 0.33 min), and their ±0.1 min identification windows did not overlap. dentification was additionally supported by the remaining transitions (*m*/*z* 288.9→92.8 and 181→152 for acrinathrin, and *m*/*z* 197→141 for cyhalothrin) and ion-ratio agreement. The risk of false-positive identification was further controlled using sequence-level reagent and matrix blanks and carryover checks. LOQ-level matrix-fortified quality-control samples included in each analytical sequence were used to verify analyte detectability and monitor the risk of false-negative findings near the reporting limit. Collectively, these controls supported the selectivity and detectability of the analytical workflow. However, because the results obtained for routine samples were not independently confirmed using a different analytical method, numerical false-positive and false-negative rates could not be reliably estimated for the real-sample dataset.

Recent advances in LC-MS/MS-based pesticide residue analysis have been focused primarily on optimizing established sample preparation strategies rather than developing fundamentally new extraction concepts. Among approximately 70 validated methods published between 2021 and 2025, QuEChERS-based workflows accounted for nearly 74% of all analytical procedures owing to their robustness, scalability, and regulatory acceptance. Nevertheless, conventional QuEChERS methods remain inadequate for highly polar pesticides and transformation products due to their poor partitioning into acetonitrile, which leads to reduced extraction efficiency, increased matrix effects, and variable recoveries. Consequently, dedicated extraction protocols and matrix-specific clean-up procedures are still required for these compounds [24,25]. The limitations of using a single multiresidue method have led to the development of complementary analytical procedures for compounds with different physicochemical properties. Robles-Molina et al. combined QuEChERS and QuPPe extraction with parallel HILIC and reversed-phase LC–MS/MS for 41 pesticides with log K_ow values ranging from −4 to +5.5. The method provided suitable retention of polar and non-polar compounds, peak-area precision below 6% RSD for most analytes, and retention-time stability within ±0.1 min. However, low or negligible recoveries were observed for some compounds at the extremes of the polarity range, showing that additional optimization may still be required [26]. Monteiro et al. developed the QuEChERSER approach, in which an acetonitrile/water extract was divided between UHPLC–MS/MS and LPGC–MS/MS analyses. In bovine muscle, 221 of 259 analytes (85%) achieved recoveries of 70–120% with interday RSDs ≤25%. Analytes that gave unsatisfactory results with one analytical technique were often successfully determined with the other [27]. The method was subsequently validated in catfish muscle, covering 231 analytes by UHPLC–MS/MS and 167 by LPGC–MS/MS, with 49 analytes included in both analyses. Recovery and precision criteria were fulfilled by 222 pesticides, metabolites, and PCBs (91%) [28]. These studies confirm that methods based on different extraction and detection procedures can complement each other and provide broader analytical coverage [26,27,28].

A similar complementarity was observed in the present study. The QuEChERS-based Method 1 provided higher recoveries for several relatively hydrophobic fluorinated pesticides, whereas the methanol-based Method 2 was more effective for selected polar compounds. For example, flazasulfuron recovery increased from 71.9 to 81.4% with Method 1 to 93.7–103% with Method 2, while penoxsulam improved from 79.5 to 86.3% to 93.2–101%. Method 2 also enabled efficient determination of the polar transformation products TFNA and TFNG, with recoveries of 93.6–99.6% and 89.0–100.9%, respectively. In contrast, mefentrifluconazole showed considerably higher recoveries with Method 1 (81.6–87.1%) than with Method 2 (41.2–47.3%), and a similar trend was observed for FM-6-1 (97.7–101% versus 48.5–58.5%). Thus, neither extraction procedure was universally superior; instead, their performance depended on the physicochemical properties of individual analytes. The combination of different extraction procedures with complementary LC–MS/MS and GC–MS/MS determination therefore represents a deliberate multi-method strategy to extend the analytical coverage. The recovery values obtained in the present study were generally comparable with, or higher than, those reported for the same pesticides in previously validated methods. For flonicamid, recoveries of 87.8, 96.7, and 97.9% were obtained in potato at fortification levels of 0.010, 0.100, and 0.400 mg kg^−1^, respectively, with corresponding RSD values of 6.6, 4.2, and 3.9%. These results agree well with the recoveries of 84.3–99.3% and RSD values of 0.41–5.95% reported for flonicamid in peach, cucumber, cabbage, and cotton using QuEChERS–UHPLC–MS/MS at three fortification levels [29]. For fludioxonil, recoveries in potato increased from 92.9% at 0.010 mg kg^−1^ to 96.9% at 0.100 mg kg^−1^ and 105% at 0.400 mg kg^−1^, while the corresponding RSD values decreased from 7.5 to 2.6–3.0%. Yao et al. reported recoveries of 81, 91, and 94% in cherry samples fortified at 0.01, 0.5, and 5 mg kg^−1^, respectively, with RSD values of 11.9, 3.7, and 2.5% [30].

The Method 2 sample-preparation procedure developed in the present study addresses these limitations by combining methanol extraction, pH adjustment (5.5–7.5), and dispersive C18 clean-up before LC-MS/MS analysis. In contrast to conventional QuEChERS protocols based on acetonitrile extraction and salt-induced phase partitioning, the proposed workflow was designed to complement the QuEChERS-based method by extending the analytical coverage to polar fluorinated pesticides and selected transformation products with diverse physicochemical properties. The adjustment of extract pH improves the compatibility of analytes with different acid-base characteristics, whereas C18 efficiently removes hydrophobic co-extractives responsible for ion suppression and matrix interference. Thus, the developed procedure complements the QuEChERS-based methods by extending the analytical coverage to polar fluorinated pesticides and selected transformation products in high-water-content food matrices while maintaining a simple and robust analytical workflow.

In the present study, the highly polar transformation products TFA and DFA were determined using methanol-based extraction. The QuPPe methodology was therefore developed specifically for highly polar pesticides. An important methodological contribution to the development of the QuPPe method was provided by Schäfer et al. (2025) [31], who demonstrated that reduced recoveries of highly polar analytes are not solely associated with the extraction solvent but are also strongly influenced by interactions with endogenous matrix components, particularly Ca^2+^ and Fe^3+^ ions. By introducing 100 mg of EDTA during QuPPe extraction, the authors successfully minimized metal–analyte complexation and improved method robustness. Furthermore, the authors demonstrated that proteins and other matrix constituents may continue to affect analyte recoveries even after metal complexation has been reduced, highlighting the complexity of matrix effects in the analysis of highly polar compounds [31].

The obtained validation results clearly demonstrate that the extraction efficiency strongly depends on the physicochemical properties of the analytes, particularly their polarity, water solubility, and hydrophobicity expressed as the octanol–water partition coefficient (logP). Such behavior is consistent with the extraction principles of both QuEChERS and methanol-based methods, which were originally designed for compounds with distinctly different physicochemical characteristics. Method 1 employs acetonitrile partitioning and is considered the reference multiresidue approach for low- and medium-polar pesticides. In contrast, Method 2 is based on methanol extraction followed by adjustment of the sample pH to 5.5–7.5 and was applied to selected polar pesticides that were not efficiently recovered using the conventional QuEChERS procedure. Having demonstrated satisfactory analytical performance, the validated methods were subsequently applied to real food samples to evaluate the occurrence and distribution of fluorinated pesticides and their transformation products.

Fludioxonil was quantified in a total of 12 samples. All positive results for this fungicide were obtained exclusively from the pear matrix. The concentrations ranged from 0.013 mg kg^−1^ to 0.22 mg kg^−1^. While 11 of these samples showed consistently low residues below 0.09 mg kg^−1^, a single pear sample exhibited a significantly higher level of 0.22 mg kg^−1^. In the present dataset, fludioxonil was detected only in pear samples; however, in the absence of crop-treatment records, no conclusions regarding commodity-specific application patterns can be drawn. The occurrence of fludioxonil exclusively in pear samples in the present dataset may reflect commodity-specific use patterns; however, this interpretation cannot be confirmed without access to crop-treatment records. Fludioxonil is a synthetic analogue of a naturally occurring antifungal compound produced by *Pseudomonas* strains. Under laboratory conditions, it does not undergo significant hydrolysis, oxidation, or other abiotic transformations, and its structure remains stable for 200–300 days. After 21 days, only approximately 5% loss of fludioxonil from solution was observed, with no release of fluoride ions. Alexandrino et al. (2020) [32] demonstrated that fludioxonil can undergo complete biodegradation and defluorination, but only in the presence of specialized microorganisms such as *Pseudomonas*, *Ochrobactrum*, and *Comamonas*. An estuarine microbial consortium achieved 100% removal and 90–100% defluorination after 63 days of enrichment, whereas a soil-derived consortium required 105 days for complete removal and 147 days for full defluorination. Kinetic studies showed that at concentrations of 1–10 mg L^−1^, complete degradation and defluorination occurred within 14–21 days, following first-order kinetics. Only at 25 mg L^−1^ was a pronounced slowdown observed—after 28 days, removal reached approximately 90%, while defluorination did not exceed 25% [32].

Flonicamid was not detected in any of the analyzed samples; instead, only its metabolites TFNA and TFNG were quantified. TFNA was quantified in seven samples, including three pear samples, two watermelon samples, one potato sample, and one onion sample, at concentrations ranging from 0.014 to 0.140 mg kg^−1^. TFNA was the most frequently detected transformation product of flonicamid. Its occurrence in different commodities is consistent with the environmental persistence of this metabolite and indicates that transformation products may remain detectable even when the parent compound is absent. TFNG was quantified in four samples. These results were detected in three different commodities: two potato samples (0.022 and 0.100 mg kg^−1^), one onion sample (0.032 mg kg^−1^), and one pear sample (0.019 mg kg^−1^). TFNG occurred less frequently than TFNA, but its detection in multiple commodities further highlights the importance of including flonicamid transformation products in routine residue monitoring. However, in the absence of crop-treatment records or direct precursor–product evidence, its occurrence cannot be unequivocally attributed to the application of flonicamid. The occurrence of TFNA and TFNG in the absence of flonicamid is consistent with the transformation pathways of flonicamid reported in the literature. In the environment, flonicamid undergoes degradation to form several metabolites, including N-(4-trifluoromethylnicotinoyl) glycine (TFNG), 4-trifluoromethylnicotinic acid (TFNA), and 4-trifluoromethylnicotinamide (TFNA-AM) [33]. The EU residue definition for flonicamid is “flonicamid (sum of flonicamid, TFNA and TFNG, expressed as flonicamid)” [34]. Flonicamid exhibits high stability under acidic conditions and at elevated temperatures but degrades rapidly under alkaline conditions and in soil. Following incubation at 54 °C for 14 weeks, 15.83% of the parent compound had degraded. The degradation half-life (DT_50_) was determined to be 252.05 days in 1.0 N HCl, 7.72 days in 0.01 N NaOH, 0.95 days in 1.0 N NaOH, and 2.46 days in clay loam soil (pH 8.59) [35]. To date, only a limited number of bacterial strains capable of degrading flonicamid have been reported. *Alcaligenes faecalis* CGMCC 17553 degraded 98.8% of FLO (209.7 mg L^−1^) within 96 h via a nitrilase-mediated pathway, producing N-(4-trifluoromethylnicotinoyl) glycinamide (TFNG-AM) and N-(4-trifluoromethylnicotinoyl)glycine (TFNG). *Ensifer adhaerens* CGMCC 6315 removed 92% of FLO (199.4 mg L^−1^) within 24 h through a nitrile hydratase (NHase)-mediated pathway, yielding TFNG-AM. *Microvirga flockulans* CGMCC 1.16731 degraded FLO via the sequential action of nitrile hydratase (NHase) and amidase, resulting in the formation of TFNG-AM followed by TFNG. *Variovorax boronicumulans* CGMCC 4969 utilized both the nitrilase and NHase/amidase pathways, producing the same metabolites. In contrast, *Ensifer meliloti* CGMCC 7333 and *Aminobacter* sp. CGMCC 1.17253 degraded Flonicamid exclusively via the NHase pathway, with TFNG-AM as the sole identified metabolite. Further hydrolysis of TFNG-AM to TFNG by amidase has been reported only for *Pseudomonas stutzeri* CGMCC 22915, which achieved approximately 60% conversion [36].

Fluazinam was successfully quantified in seven samples. These results were distributed across three different food matrices, including three pear samples, three potato samples, and one carrot sample. The concentrations across these matrices ranged from 0.014 mg kg^−1^ to 0.082 mg kg^−1^, with the maximum value detected in a pear extract (0.082 mg kg^−1^). The persistence of fluazinam in the analyzed samples, despite its known susceptibility to hydrolysis under alkaline conditions, likely reflects the predominantly neutral to slightly acidic environments of the examined food matrices, where degradation proceeds much more slowly, resulting in measurable residues at harvest and underscoring the need for continued monitoring to ensure food safety. The primary product of fluazinam hydrolysis is 5-chloro-6-(3-chloro-2,6-dinitro-4-trifluoromethylanilino)nicotinic acid (CAPA), which is subsequently degraded to 6-(4-carboxy-3-chloro-2,6-dinitroanilino)-5-chloronicotinic acid (DCPA). In addition to hydrolysis, biodegradation and photodegradation also contribute to the dissipation of fluazinam in the environment; however, their roles are considered less significant than that of hydrolytic degradation [37]. Fluazinam is degraded primarily through hydrolysis and aerobic microbial biodegradation in soil. Its dissipation is generally described by first-order (FO) kinetics, with the degradation rate being strongly influenced by environmental pH, soil properties, temperature, and moisture content. Under laboratory conditions, the degradation half-life (DT_50_) in soils ranged from 26 to 54 days, with the greatest persistence observed in lateritic soil and the most rapid degradation occurring in black soil. In aquatic environments, the stability of fluazinam is highly pH-dependent. Under acidic conditions (pH 4), the DT_50_ was approximately 120–144 days, decreasing to 30–33 days at neutral pH, and further to approximately 2–8 days under alkaline conditions (pH 9.0–9.2). These findings indicate that hydrolysis is markedly accelerated in the presence of hydroxide ions, making it the dominant degradation process under alkaline conditions [37]. Fluazinam exhibits high persistence in soil, and its degradation is strongly influenced by environmental conditions. The highest degradation rate was observed immediately after application, followed by a gradual decline over time. The degradation half-life (DT_50_), defined as the time required for the concentration of the parent compound to decrease by 50%, ranged from 355 to 583 days under variable field conditions and from 418 to 833 days under controlled laboratory conditions. Soil organic matter accelerated fluazinam degradation by enhancing microbial activity. Owing to its high persistence, fluazinam may remain in soil for multiple growing seasons, highlighting its long-term environmental persistence [38].

Fluopyram was detected in three samples, including pear and potato, with concentrations ranging from 0.016 to 0.082 mg kg^−1^. The occurrence of fluopyram in different food matrices indicates that this compound can remain on crops until harvest. These findings highlight the relevance of systematic monitoring of pesticide residues in food, which supports transparent control of agricultural practices and contributes to maintaining high standards of food safety. In the environment, fluopyram is degraded through microbial biodegradation. The degradation process is initiated by hydroxylation to form fluopyram-7-hydroxy, which is subsequently transformed into fluopyram benzamide (BZM), pyridylcarboxylic acid (PCA), and the methyl sulfoxide metabolite. The final stage of degradation is mineralization, resulting in the formation of carbon dioxide (CO_2_) and non-extractable soil-bound residues. Studies have shown that the maximum proportions of the identified metabolites reached 4.2% for fluopyram-7-hydroxy, 1.1% for BZM, 0.7% for PCA, and 1.0% for the methyl sulfoxide metabolite [39,40]. Microbial degradation of fluopyram by *Bacillus subtilis* was limited, reaching approximately 2% after 2 and 5 days of incubation and 7.5% after 7 and 14 days. In contrast, *Trichoderma harzianum* exhibited the highest degradation efficiency, achieving fluopyram degradation rates ranging from 74.3% to 81.7% [41].

Flupyradifurone was detected only once, in a pear sample at 0.041 mg kg^−1^, indicating that this compound appeared sporadically in the analyzed dataset. Such isolated findings show that individual pesticides can occur unevenly across crops and seasons, reinforcing the importance of continued monitoring to capture both frequent and infrequent residue occurrences in food products. The very low frequency of quantification suggests that flupyradifurone was either rarely applied or degraded effectively below the reporting limits in the other investigated crops. Flupyradifurone undergoes environmental transformation, resulting in the formation of several degradation products. The major and most persistent metabolite is difluoroacetic acid (DFA). Additional transformation products include 6-chloronicotinic acid (6-CNA), formed through cleavage of the chloropyridine moiety, and DFEAF (difluoroethylamino-furanone), which originates from transformation of the furanone moiety. In plants, hydroxylated metabolites of flupyradifurone and their conjugates have also been identified. In field trials on tomatoes treated at an application rate of 200 g ha^−1^, the initial flupyradifurone residue was 3.15 mg kg^−1^ at 2 h after application and declined to 0.04 mg kg^−1^ after 28 days [42,43].

Difluoroacetic acid (DFA) and trifluoroacetic acid (TFA) are persistent fluorinated acids that may be formed through the transformation of several fluorinated pesticides and other fluorinated substances. DFA was quantified in five pear samples at concentrations ranging from 0.022 to 0.350 mg kg^−1^. The occurrence of DFA is consistent with its known role as a transformation product of selected fluorinated pesticides. Nevertheless, because crop-treatment records and direct precursor–product evidence were not available, the detected DFA cannot be unequivocally linked to the application or degradation of any specific active substance and other environmental sources cannot be excluded. TFA was quantified in nine samples, including three carrot samples, three potato samples, two cauliflower samples, and one onion sample, at concentrations ranging from 0.072 to 0.190 mg kg^−1^. Because TFA may originate from multiple environmental and anthropogenic sources, including the transformation of several fluorinated pesticides, its occurrence in the analysed commodities should not be interpreted as direct evidence of the application or degradation of any specific pesticide. The occurrence of persistent fluorinated acids, even in samples where the corresponding parent pesticides were not detected, demonstrates the importance of monitoring both parent compounds and transformation products to obtain a more comprehensive picture of fluorinated pesticide residues in food. These findings highlight the importance of monitoring not only parent compounds but also their persistent transformation products, which may provide a more complete picture of pesticide fate in agricultural systems.

## 5. Conclusions

This study demonstrates that comprehensive determination of fluorinated pesticides and their transformation products requires complementary extraction approaches to ensure adequate coverage of compounds with diverse physicochemical properties. Extraction efficiency was primarily governed by analyte polarity and lipophilicity, with methanol-based extraction providing superior performance for highly polar compounds, including sulfonylurea herbicides and the ultrashort-chain transformation products TFNA and TFNG. Application of the validated methods to real food samples confirmed the occurrence of both parent fluorinated pesticides and persistent transformation products. Nine target analytes were detected in the 132 analysed commercial food samples, including five parent pesticides and four transformation products. The detection of TFNA, TFNG, DFA, and TFA demonstrates that monitoring parent compounds alone may underestimate the overall burden of fluorinated residues in food. The proposed analytical strategy expands the scope of fluorinated pesticide residue analysis. It supports more comprehensive food-safety monitoring through the complementary determination of parent compounds and environmentally persistent transformation products using several extraction and instrumental methods. The developed analytical workflow represents a practical tool for routine food monitoring and may support future surveillance programs focused on fluorinated pesticides and PFAS-related transformation products. Considering the increasing use of fluorinated pesticides and the growing concern regarding persistent fluorinated transformation products, the implementation of such complementary analytical approaches will be essential for future food-safety monitoring and exposure assessment.

## Figures and Tables

**Table 1:** Validation results for pesticide residues obtained in method 1, based on the QuEChERS procedure applied to high-water-content foods (potato) in LC-MS/MS, including determination of recovery (Rec), RSD, and expanded uncertainty (Uexp).

Compounds	LOQs [mg kg−1]	1∙LOQs			10∙LOQs			40∙LOQs			ME [%]
		Rec [%]	RSD [%]	Uexp [%]	Rec [%]	RSD [%]	Uexp [%]	Rec [%]	RSD [%]	Uexp [%]	
Beflubutamid	0.010	94.6	7.2	21.8	97.9	2.6	8.2	95.1	2.9	12.5	94.6
Benzobicyclon	0.010	101	4.0	10.8	92.1	1.8	16.4	94.5	0.9	11.3	78.6
Chlorfluazuron	0.010	95.3	1.5	10.2	96.5	1.9	8.6	98.6	1.2	4.2	333.3
Cyflumetofen	0.010	96.1	6.2	18.1	80.2	7.0	43.2	103	5.5	16.1	99.5
Diflubenzuron	0.010	101	2.3	6.4	99.1	1.7	4.9	93.9	2.7	14.2	7.4
Epoxiconazole	0.010	100	4.8	12.8	98.2	4.3	12.0	97.5	2.7	8.8	74.3
Flazasulfuron	0.010	81.4	1.4	37.3	71.9	1.9	4.1	80.9	2.4	38.6	2.3
Florasulam	0.010	79.7	3.8	8.3	78.4	2.8	6.2	86.7	3.4	28.1	34.4
Fluazinam	0.010	97.6	1.9	6.9	95.0	2.4	11.7	97.0	2.5	8.9	51.3
Flubendiamide	0.010	93.8	2.0	13.4	96.4	1.6	8.5	97.4	0.5	5.3	12.3
Fluquinconazole	0.010	100	6.9	18.7	94.8	1.5	11.2	97.4	1.4	6.4	74.4
Flufenoxuron	0.010	94.6	2.6	12.8	91.5	3.1	18.7	97.5	2.3	7.8	61.9
Fluopicolide	0.010	89.0	2.0	22.5	88.9	2.2	22.9	94.8	3.2	13.3	798.9
Fluopyram benzamide (M25)	0.010	91.3	4.1	20.3	92.9	1.6	14.7	95.9	0.9	8.6	52.2
Flupyradifurone	0.010	90.0	4.8	23.4	96.0	1.9	9.4	103	2.5	9.0	94.7
Flutianil	0.010	88.8	3.4	24.1	85.9	1.2	28.4	96.8	3.2	10.7	1.0
Flutolanil	0.0050	98.7	3.0	8.4	96.3	2.6	10.1	93.8	1.6	13.1	6003.3
Fluxapyroxad	0.010	95.4	2.0	10.6	92.3	1.8	16.1	102	2.4	7.2	1691.2
Hexaflumuron	0.010	99.7	1.7	4.5	98.1	1.5	5.4	97.8	0.7	4.7	65.8
Hydramethylnon	0.010	90.5	1.2	19.3	91.6	0.3	16.9	95.6	1.3	9.4	535.6
Lufenuron	0.010	98.8	2.6	7.4	95.9	2.5	10.6	100	0.6	1.9	46.5
Mefentrifluconazole	0.010	81.6	1.2	36.9	85.3	0.8	29.4	87.1	1.1	25.9	35.9
Metaflumizone	0.010	87.0	3.7	27.7	89.2	2.0	22.1	97.4	4.0	11.9	7.8
Norflurazon	0.010	101	1.7	4.9	96.1	2.2	9.7	96.9	1.6	7.6	239.7
Novaluron	0.010	83.2	6.4	37.1	94.4	7.4	22.5	94.3	3.6	14.8	87.9
Oxathiapiprolin	0.010	95.8	4.5	14.6	98.3	1.8	5.8	98.2	3.0	8.7	32.9
Penflufen	0.010	91.8	3.4	18.5	94.8	1.3	11.0	96.4	1.2	7.9	2.0
Penoxsulam	0.010	79.5	1.7	3.8	72.0	0.5	1.2	86.3	1.6	27.8	5.7
Prosulfuron	0.010	84.6	2.3	31.3	82.2	2.8	36.4	89.7	3.4	22.3	73.6
Pydiflumetofen	0.010	92.4	5.3	20.5	94.5	1.2	11.5	97.5	1.5	6.4	48.5
Sulfoxaflor	0.010	98.8	3.2	9.0	96.4	2.1	9.1	99.4	0.9	2.7	59.1
Tau-fluvalinate	0.010	82.8	7.5	39.2	86.3	3.8	29.1	97.8	4.8	13.5	95.4
Tetraconazole	0.010	101	3.5	9.6	100	2.3	6.2	98.9	0.6	2.8	78.9
Tetraniliprole	0.010	75.0	13.1	28.7	88.5	1.6	23.3	95.1	1.5	10.6	21.8
Triflumizole	0.010	88.2	3.7	25.4	89.6	1.7	21.3	92.8	2.9	16.1	290.8
Triflumizole metabolite FM-6-1	0.010	97.7	4.0	11.5	98.5	1.3	4.6	101	1.0	3.6	15.0
Triflumuron	0.010	98.8	3.2	8.8	96.2	2.0	9.3	96.8	2.1	8.5	2381.4
Tritosulfuron	0.010	94.1	2.9	14.0	92.9	1.0	14.5	94.5	2.1	12.3	67.6

**Table 2:** Validation results for pesticide residues obtained in method 1, based on the QuEChERS procedure applied to high-water-content foods (potato) in GC-MS/MS, including determination of recovery (Rec), RSD, and expanded uncertainty (Uexp).

Compounds	LOQs [mg kg^−1^]	1∙LOQs			10∙LOQs			40∙LOQs			ME [%]
		Rec [%]	RSD [%]	Uexp [%]	Rec [%]	RSD [%]	Uexp [%]	Rec [%]	RSD [%]	Uexp [%]	
Acrinathrin	0.010	89.0	6.2	27.1	101	3.0	8.3	106	2.7	14.2	34.7
Benfluralin	0.010	81.7	14.4	31.6	97.5	3.2	10.0	99.5	2.0	5.4	27.3
Bifenthrin	0.010	83.4	2.9	33.9	99.9	5.2	14.0	107	3.3	17.0	42.8
Chlorfenapyr	0.010	70.5	7.6	16.7	106	3.0	15.2	106	3.3	15.1	31.5
Cyflufenamid	0.010	87.0	11.6	39.3	98.1	3.0	8.9	108	1.5	16.0	25.9
Cyhalothrin (sum of isomers)	0.010	89.1	3.3	23.4	101	2.0	6.2	107	2.7	15.1	48.6
Diflufenican	0.010	88.2	4.8	26.6	105	2.7	12.8	105	3.6	14.1	36.2
Fipronil	0.0020	101	15.5	41.7	84.0	7.6	37.2	97.3	7.8	21.4	7.0
Fipronil sulfone	0.0030	86.8	19.8	43.4	91.6	2.8	18.3	104	4.9	15.4	33.8
Flonicamid	0.010	87.8	6.6	29.6	96.7	4.2	12.9	97.9	3.9	11.1	21.7
Fludioxonil	0.010	92.9	7.5	24.3	96.9	2.6	9.2	105	3.0	12.6	38.5
Fluensulfone	0.010	63.9	6.8	14.8	96.1	2.8	10.8	96.6	1.5	7.7	24.6
Flufenacet	0.010	95.6	7.3	21.2	101	2.0	5.8	105	2.5	11.8	30.8
Flumetralin	0.010	75.1	6.8	14.8	75.0	5.7	16.1	69.8	5.2	14.6	41.3
Fluopyram	0.010	87.6	2.7	25.8	97.2	4.1	12.1	106	2.4	13.3	28.9
Flurochloridone	0.010	94.9	9.7	27.3	115	2.4	30.3	105	3.8	14.9	35.6
Flutolanil	0.010	95.2	2.6	11.9	103	1.3	7.3	107	1.9	15.9	5.5
Tau-fluvalinate	0.010	83.0	4.8	36.0	102	3.2	9.3	106	3.2	14.7	56.4
Indoxacarb	0.010	100	9.4	25.2	101	4.3	11.7	105	3.2	13.9	44.1
Isopyrazam	0.010	75.0	10.6	23.2	103	6.9	20.0	95.7	4.2	14.0	32.7
Oxyfluorfen	0.010	94.9	10.6	29.7	102	9.1	25.1	107	2.8	16.9	11.2
Penthiopyrad	0.010	90.7	18.6	40.8	97.6	3.8	11.3	104	4.8	15.7	37.2
Picoxystrobin	0.010	92.4	5.1	20.2	96.4	2.3	9.6	107	2.9	16.2	29.8
Picolinafen	0.010	82.2	7.6	40.2	101	4.3	11.5	107	3.8	17.0	34.1
Pyridalyl	0.010	84.6	3.8	32.3	103	3.4	10.4	108	3.0	17.3	52.3
Tefluthrin	0.010	83.1	5.3	36.3	103	2.2	8.2	108	2.0	16.2	39.6
Trifloxystrobin	0.010	86.6	2.7	27.6	101	1.9	5.6	105	2.0	11.9	31.4
Triflumizole	0.010	85.0	4.0	31.6	97.1	3.2	10.2	105	3.1	13.7	27.8

**Table 3:** Validation results for pesticide residues obtained in method 2, applied to high-water-content foods (potato) in LC-MS/MS, including determination of recovery (Rec), RSD, and expanded uncertainty (Uexp).

Compounds	LOQs [mg kg^−1^]	1∙LOQs			10∙LOQs			40∙LOQs			ME [%]
		Rec [%]	RSD [%]	Uexp [%]	Rec [%]	RSD [%]	Uexp [%]	Rec [%]	RSD [%]	Uexp [%]	
Flazasulfuron	0.010	93.7	1.0	12.9	101.7	1.2	4.7	103	1.9	7.6	2.3
Flonicamid	0.010	90	2.2	21.2	102.3	0.8	5.1	99.9	1.7	4.6	19.2
Fluazaindolizine	0.010	75.9	4.6	13.0	88.8	3.6	24.3	90.5	2.7	20.2	11.6
Fludioxonil metabolite CGA-192155	0.010	93.1	1.9	14.7	100.9	2.1	5.9	101	2.1	6.3	28.1
Fluometuron	0.010	84	5.4	34.2	93.1	1.4	14.2	95.3	2.6	11.6	35.5
Fluopyram benzamide (M25)	0.010	97	1.7	7.3	106.7	1.9	14.4	103	1.1	6.0	52.2
Isoxaflutole	0.010	62.2	4.5	12.7	72.7	3.0	8.4	70.4	4.3	12.0	12.0
Isoxaflutole diketonitrile	0.010	95.6	4.1	14.0	103.3	2.3	9.2	99.6	2.1	5.6	0.8
Mefentrifluconazole	0.010	41.2	14.1	39.9	40.3	0.5	1.3	47.3	2.9	8.1	35.9
Penoxsulam	0.010	93.2	2.9	15.5	100.4	1.4	3.8	101	2.7	7.6	5.7
Prosulfuron	0.010	81	2.7	38.2	96.3	4.6	14.2	96.9	2.3	8.7	12.1
Pyroxsulam	0.010	85.5	1.2	29.1	97.9	2.0	6.7	98.0	2.9	8.8	15.7
Tetraniliprole	0.010	84.0	2.7	32.8	89.0	1.6	22.3	89.3	2.1	22.0	21.8
TFNA (4-(trifluoromethyl)nicotinic acid)	0.010	93.6	9.5	27.9	99.6	2.1	5.6	98.7	1.5	4.7	12.3
TFNG (N-(4-trifluoromethylnicotinoyl)glycine)	0.010	89.0	2.5	22.9	100.9	2.7	7.4	98	3.4	9.7	12.5
Triflumizole metabolite FM-6-1	0.010	48.5	9.2	26.2	56.0	3.5	10.0	58.5	4.4	12.4	18.2
Triflusulfuron metabolite IN-M72222	0.010	90.9	1.2	18.6	102.4	1.0	5.5	98.5	1.4	4.9	35.8
Tritosulfuron	0.010	81.7	4.4	38.2	96.3	2.3	9.4	101	1.1	3.2	12.3
Tritosulfuron metabolite AMTT	0.010	99.3	3.9	10.5	104.3	1.8	10.0	103	0.9	5.6	46.9

**Table 4:** Validation results for pesticide residues obtained in method 3, based on the QuPPe procedure applied to high-water-content foods (potato) in LC-MS/MS, including determination of recovery (Rec), RSD, and expanded uncertainty (Uexp).

Compounds	LOQs [mg kg^−1^]	1∙LOQs			10∙LOQs			40∙LOQs			ME [%]
		Rec [%]	RSD [%]	Uexp [%]	Rec [%]	RSD [%]	Uexp [%]	Rec [%]	RSD [%]	Uexp [%]	
Difluoroacetic acid (DFA)	0.020	90.9	7.4	26.5	107.0	6.1	22.7	109.0	2.0	18.9	36.9
Trifluoroacetic acid (TFA)	0.050	105.1	3.0	13.0	109.0	3.1	19.4	105.0	1.9	11.3	49.3

**Table 5:** Occurrence and concentrations of fluorinated pesticides and selected transformation products detected in commercial food samples. * Compliance with the applicable MRLs was assessed taking into account the expanded measurement uncertainty determined by the laboratory (U = 50%, k = 2), in accordance with the laboratory decision rule. ** MRL refers to the residue definition “flonicamid (sum of flonicamid, TFNA and TFNG expressed as flonicamid)”. Compliance was assessed after conversion of TFNA and TFNG to flonicamid equivalents using conversion factors of 1.20 and 0.92, respectively, and comparison of the calculated residue-definition result with the applicable MRL.

No.	Sample ID	Analyte	Concentration [mg kg^−1^]	Commodity	MRL [mg kg^−1^]	LOQ [mg kg^−1^]	Compliance
1	42	Fluazinam	0.014	Carrot	0.01	0.010	Compliant *
2	624	Fluazinam	0.027		0.01	0.010	Non-compliant
3	111	TFA	0.180		Not established	0.050	Not applicable
4	382	TFA	0.100		Not established	0.050	Not applicable
5	624	TFA	0.080		Not established	0.050	Not applicable
6	580	TFA	0.170	Cauliflower	Not established	0.050	Not applicable
7	703	TFA	0.110		Not established	0.050	Not applicable
8	480	TFNA	0.022	Onion	0.03 **	0.010	Compliant *
9	480	TFNG	0.032		0.03 **	0.010	Compliant *
10	614	TFA	0.100		Not established	0.050	Not applicable
11	370	Fluazinam	0.016	Potato	0.02	0.010	Compliant
12	326	Fluazinam	0.022		0.02	0.010	Compliant *
13	415	Fluopicolide	0.018		0.030	0.010	Compliant
14	323	TFNA	0.032		0.2 **	0.010	Compliant
15	323	TFNG	0.022		0.2 **	0.010	Compliant
16	370	TFNG	0.100		0.2 **	0.010	Compliant
17	415	TFA	0.072		Not established	0.050	Not applicable
18	424	TFA	0.077		Not established	0.050	Not applicable
19	326	TFA	0.190		Not established	0.050	Not applicable
20	415	Fluopyram	0.016		0.08	0.010	Compliant
21	424	Fluopyram	0.022		0.08	0.010	Compliant
22	78	Fludioxonil	0.025	Pear	5	0.010	Compliant
23	96	Fludioxonil	0.035		5	0.010	Compliant
24	85	Fludioxonil	0.016		5	0.010	Compliant
25	161	Fludioxonil	0.014		5	0.010	Compliant
26	216	Fludioxonil	0.019		5	0.010	Compliant
27	280	Fludioxonil	0.086		5	0.010	Compliant
28	319	Fludioxonil	0.026		5	0.010	Compliant
29	445	Fludioxonil	0.014		5	0.010	Compliant
30	508	Fludioxonil	0.020		5	0.010	Compliant
31	640	Fludioxonil	0.220		5	0.010	Compliant
32	66	Fludioxonil	0.020		5	0.010	Compliant
33	714	Fludioxonil	0.013		5	0.010	Compliant
34	623	Flupyradifurone	0.041		0.6	0.010	Compliant
35	114	Fluazinam	0.016		0.3	0.010	Compliant
36	280	Fluazinam	0.014		0.3	0.010	Compliant
37	623	Fluazinam	0.082		0.3	0.010	Compliant
38	18	TFNA	0.014		0.3 **	0.010	Compliant
39	331	TFNA	0.022		0.3 **	0.010	Compliant
40	623	TFNA	0.033		0.3 **	0.010	Compliant
41	18	TFNG	0.019		0.3 **	0.010	Compliant
42	132	DFA	0.028		0.2	0.020	Compliant
43	209	DFA	0.045		0.2	0.020	Compliant
44	331	DFA	0.110		0.2	0.020	Compliant
45	445	DFA	0.022		0.2	0.020	Compliant
46	623	DFA	0.350		0.2	0.020	Compliant *
47	566	Fluopyram	0.082		0.8	0.010	Compliant
48	700	TFNA	0.024	Watermelon	0.4 **	0.010	Compliant
49	701	TFNA	0.140		0.4 **	0.010	Compliant

## Data Availability

The original contributions presented in this study are included in the article/Supplementary Material. Further inquiries can be directed to the corresponding authors.

## Supplementary Materials

The following supporting information can be downloaded at: https://www.mdpi.com/article/10.3390/foods15173050/s1, Table S1. List of pesticides, cas number and concentration in stock solution; Table S2. List of pesticides, along with total retention time (TR) [min], precursor [*m*/*z*], product [*m*/*z*], fragmentor, CAV [V] and ion transitions with collision energy—CE [eV], and polarity for method 1 based in QuEChERS for LC-MS/MS; Table S3. List of pesticides, along with total retention time (TR) [min], precursor [*m*/*z*], product [*m*/*z*], fragmentor, CAV [V] and ion transitions with collision energy—CE [eV], and polarity for method 1 based in QuEChERS method for GC-MS/MS; Table S4. List of pesticides, along with total retention time (TR) [min], precursor [*m*/*z*], product [*m/z*], fragmentor, CAV [V] and ion transitions with collision energy—CE [eV], and polarity for method 2 for LC-MS/MS; Table S5. List of pesticides, along with total retention time (TR) [min], precursor [*m*/*z*], product [*m*/*z*], fragmentor, CAV [V] and ion transitions with collision energy—CE [eV], and polarity for method 1 based in QuPPe method for LC-MS/MS; Table S6. LogP values of the pesticides and pesticide metabolites included in the study; Figure S1. Representative MRM chromatograms of DFA: (A) reagent blank; (B) blank pear-matrix extract; and (C) pear matrix-matched calibration standard at the validated LOQ of 0.020 mg kg^−1^; Figure S2. Representative MRM chromatograms of TFA: (A) reagent blank; (B) blank pear-matrix extract; and (C) pear matrix-matched calibration standard at the validated LOQ of 0.050 mg kg^−1^.
