# Supplementary material for: Determination of Fluorinated Pesticide Residues and Their Selected Transformation Products in High-Water-Content Foods Using Complementary LC–MS/MS and GC–MS/MS Methods
Source: Foods. 2026 Aug 28;15(17):3050. doi: 10.3390/foods15173050 (PMC13564678; doi:10.3390/foods15173050)

Table S1. List of pesticides, cas number and concentration in stock solution.

MIX 1 LC		
**Name of compound**	**CAS Numbe**	**Concentration in stock [µg/mL]**
Beflubutamid	113614-08-7	10
Benzobicyclon	156963-66-5	10
Chlorfluazuron	71422-67-8	10
Cyflumetofen	400882-07-7	10
Diflubenzuron	35367-38-5	10
Epoxiconazole	133855-98-8	10
Flazasulfuron	104040-78-0	10
Florasulam	145701-23-1	10
Fluazinam	79622-59-6	10
Flubendiamide	272451-65-7	10
Fluquinconazole	136426-54-5	10
Flufenoxuron	101463-69-8	10
Fluopicolide	239110-15-7	10
Fluopyram benzamide (M25)	360-64-5	10
Flupyradifurone	951659-40-8	10
Flutianil	958647-10-4	10
Flutolanil	66332-96-5	10
Fluxapyroxad	907204-31-3	10
Hexaflumuron	86479-06-3	10
Hydramethylnon	67485-29-4	10
Lufenuron	103055-07-8	10
Mefentrifluconazole	1417782-03-6	10
Metaflumizone	139968-49-3	10
Norflurazon	27314-13-2	10
Novaluron	116714-46-6	10
Oxathiapiprolin	1003318-67-9	10
Penflufen	494793-67-8	10
Penoxsulam	219714-96-2	10
Prosulfuron	94125-34-5	10
Pydiflumetofen	1228284-64-7	10
Sulfoxaflor	946578-00-3	10
Tau-fluvalinate	102851-06-9	10
Tetraconazole	112281-77-3	10
Tetraniliprole	1229654-66-3	10
Triflumizole	68694-11-1	10
Triflumizole metabolite FM-6-1	109849-99-2	10

Triflumuron	64628-44-0	10
Tritosulfuron	142469-14-5	10
**MIX1 GC**		
**Name of compound**	**CAS Numbe**	**Concentration in stock [µg/mL]**
Acrinathrin	101007-06-1	10
Benfluralin	1861-40-1	10
Bifenthrin	82657-04-3	10
Chlorfenapyr	122453-73-0	10
Cyflufenamid	180409-60-3	10
Cyhalothrin (sum of isomers)	68085-85-8	10
Diflufenican	83164-33-4	10
Fipronil	120068-37-3	10
Fipronil sulfone	120068-36-2	10
Flonicamid	158062-67-0	10
Fludioxonil	131341-86-1	10
Fluensulfone	318290-98-1	10
Flufenacet	142459-58-3	10
Flumetralin	62924-70-3	10
Fluopyram	658066-35-4	10
Flurochloridone	61213-25-0	10
Flutolanil	66332-96-5	10
Tau-fluvalinate	102851-06-9	10
Indoxacarb	144171-61-9	10
Isopyrazam	881685-58-1	10
Oxyfluorfen	42874-03-3	10
Penthiopyrad	183675-82-3	10
Picoxystrobin	117428-22-5	10
Picolinafen	137641-05-5	10
Pyridalyl	179101-81-6	10
Tefluthrin	79538-32-2	10
Trifloxystrobin	141517-21-7	10
Triflumizole	68694-11-1	10
Trifluralin	1582-09-8	10
**MIX2 LC**		
**Name of compound**	**CAS Numbe**	**Concentration in stock [µg/mL]**
Flazasulfuron	104040-78-0	10
Flonicamid	158062-67-0	10
Fluazaindolizine	1254304-22-7	10
Fludioxonil metabolite CGA-192155	126120-85-2	10
Fluometuron	2164-17-2	10

Fluopyram benzamide (M25)	360-64-5	10
Isoxaflutole	141112-29-0	10
Isoxaflutole diketonitrile	143701-75-1	10
Mefentrifluconazole	1417782-03-6	10
Penoxsulam	219714-96-2	10
Prosulfuron	94125-34-5	10
Pyroxsulam	422556-08-9	10
Tetraniliprole	1229654-66-3	10
TFNA (4-(trifluoromethyl)nicotinic acid)	158063-66-2	10
TFNG (N-(4-trifluoromethylnicotinoyl)glycine)	207502-65-6	10
Triflumizole metabolite FM-6-1	109849-99-2	10
Triflusulfuron metabolite IN-M72222	1418095-28-9	10
Tritosulfuron	142469-14-5	10
Tritosulfuron metabolite AMTT	531105-7	10
**MIX3 LC**		
**Name of compound**	**CAS Numbe**	**Concentration in stock [µg/mL]**
Difluoroacetic acid (DFA)	381-73-7	10
Trifluoroacetic acid (TFA)	76-05-1	25

Table S2. List of pesticides, along with total retention time (TR) [min], precursor [*m/z*], product [*m/z*],, fragmentor, CAV [V] and ion transitions with collision energy - CE [eV], and polarity for method 1 based in QuEChERS for LC-MS/MS.

Compound name	Precursor *m/z*	Product *m/z*	RT (min)	Fragmentor (V)	CAV (V)	CE (V)	Polarity
Beflubutamid	356.1	221	16.6	145	2	24	Positive
Beflubutamid	356.1	162.1	16.6	145	2	24	Positive
Beflubutamid	356.1	91.1	16.6	145	2	28	Positive
Benzobicyklon	446.9	411	9.6	135	5	20	Positive
Benzobicyklon	446.9	349	9.6	135	5	38	Positive
Benzobicyklon	446.9	257.2	9.6	135	5	25	Positive
Benzobicyklon	446.9	229.2	9.6	135	5	38	Positive
Chlorfluazuron	542	384.8	21.3	110	4	20	Positive
Chlorfluazuron	539.9	382.9	21.3	105	4	16	Positive
Chlorfluazuron	539.9	158	21.3	105	4	20	Positive
Cyflumetofen	465	249	20.4	98	4	5	Positive
Cyflumetofen	465	173	20.4	98	4	21	Positive
Diflubenzuron	311	158	15.25	80	7	8	Positive
Diflubenzuron	311	141	15.25	80	7	36	Positive
Epoxiconazole	330.1	121	14.5	100	3	16	Positive
Epoxiconazole	330.1	101.2	14.5	100	3	52	Positive
Flazasulfuron	408.1	181.7	12.17	121	5	16	Positive
Flazasulfuron	408.1	145.7	12.17	121	5	48	Positive

Flazasulfuron	408.1	138.7	12.17	121	5	44	Positive
Flazasulfuron	408.1	82.9	12.17	121	5	56	Positive
Florasulam	360	192	9.6	120	3	12	Positive
Florasulam	360	144	9.6	120	3	20	Positive
Florasulam	360	129	9.6	120	3	28	Positive
Fluazinam	462.9	416	19.8	110	2	10	Negative
Fluazinam	462.9	398	19.8	110	2	9	Negative
Flubendiamid	408	273.9	17	86	7	9	Positive
Flubendiamid	408	147	17	86	7	37	Positive
Flufenoxuron	489	158	20.6	70	7	25	Positive
Flufenoxuron	489	141	20.6	70	7	25	Positive
Fluopicolide	383	173	15.1	88	7	17	Positive
Fluopicolide	383	145	15.1	88	7	53	Positive
Fluopyram benzamide (M25)	190.1	170	4.9	105	5	20	Positive
Fluopyram benzamide (M25)	190.1	150	4.9	105	5	20	Positive
Fluopyram benzamide (M25)	190.1	129.8	4.9	105	5	20	Positive
Fluopyram benzamide (M25)	190.1	101.9	4.9	105	5	36	Positive
Fluopyram benzamide (M25)	190.1	75	4.9	105	5	56	Positive
Fluopyram benzamide (M25)	190.1	51.1	4.9	105	5	64	Positive
Flupyradifurone	289	126	7.9	104	4	20	Positive
Flupyradifurone	289	90	7.9	104	4	52	Positive
Flupyradifurone	289	73	7.9	104	4	88	Positive
Fluquinconazole	376	349	14.7	120	4	20	Positive
Fluquinconazole	376	307.1	14.7	120	4	24	Positive
Flutianil	427	201	17.9	90	5	37	Positive
Flutianil	427	192	17.9	90	5	28	Positive
Flutianil	427	132	17.9	90	5	61	Positive
Flutolanil	324	262	15.6	102	7	12	Positive
Flutolanil	324	242	15.6	102	7	24	Positive
Fluxapyroxad	382.1	342	14.9	113	4	20	Positive
Fluxapyroxad	382.1	314	14.9	113	4	24	Positive
Hexaflumuron	459	439	18.3	73	4	9	Negative
Hexaflumuron	459	276	18.3	73	4	17	Negative
Hydramethylnon	495.2	368	18.4	220	2	48	Positive
Hydramethylnon	495.2	323.1	18.4	220	2	28	Positive
Hydramethylnon	495.2	171	18.4	220	2	48	Positive
Lufenuron	509	325.9	19.9	94	4	17	Negative
Lufenuron	509	174.9	19.9	94	4	41	Negative
Mefentrifluconazole	398.1	182	15.75	137	5	32	Positive
Mefentrifluconazole	398.1	167.1	15.75	137	5	64	Positive
Mefentrifluconazole	398.1	133.1	15.75	137	5	92	Positive
Mefentrifluconazole	398.1	70.2	15.75	137	5	24	Positive
Metaflumizone	507.1	287.1	20	150	2	24	Positive
Metaflumizone	507.1	178	20	150	2	28	Positive
Norflurazon	304	284.1	11.8	138	5	23	Positive
Norflurazon	304	160	11.8	138	5	34	Positive
Novaluron	493	158.1	18.9	90	3	16	Positive
Novaluron	493	141.1	18.9	90	3	56	Positive
Oxathiapiprolin	540.1	500.1	15.8	125	5	28	Positive
Oxathiapiprolin	540.1	163	15.8	125	5	58	Positive
Oxathiapiprolin	540.1	139	15.8	125	5	48	Positive

Penflufen	318	234	15.7	112	4	13	Positive
Penflufen	318	141	15.7	112	4	29	Positive
Penoxsulam	484.1	483.1	12.25	169	5	1	Positive
Penoxsulam	484.1	443.2	12.25	169	5	24	Positive
Penoxsulam	484.1	326.1	12.25	169	5	40	Positive
Penoxsulam	484.1	194.7	12.25	169	5	28	Positive
Penoxsulam	484.1	166	12.25	169	5	40	Positive
Penoxsulam	484.1	164.1	12.25	169	5	40	Positive
Penoxsulam	484.1	123.8	12.25	169	5	60	Positive
Prosulfuron	420.1	167	13.1	120	3	10	Positive
Prosulfuron	420.1	141.1	13.1	120	3	15	Positive
Pydiflumetofen	425.9	406.1	17.3	135	5	11	Positive
Pydiflumetofen	425.9	193	17.3	135	5	34	Positive
Pydiflumetofen	425.9	186.2	17.3	135	5	21	Positive
Pydiflumetofen	425.9	171.1	17.3	135	5	18	Positive
Pydiflumetofen	425.9	406.1	10.03	135	5	11	Positive
Pydiflumetofen	425.9	193	10.03	135	5	34	Positive
Pydiflumetofen	425.9	186.2	10.03	135	5	21	Positive
Pydiflumetofen	425.9	171.1	10.03	135	5	18	Positive
Sulfoxaflor	276	261	8.2	86	4	8	Negative
Sulfoxaflor	276	213	8.2	86	4	12	Negative
Sulfoxaflor	276	212	8.2	86	4	12	Negative
Tau-fluvalinate	503.1	208.1	23.8	80	3	15	Positive
Tau-fluvalinate	503.1	181.1	23.8	80	3	25	Positive
Tetraconazole	372	159	15	130	2	36	Positive
Tetraconazole	372	70	15	130	2	20	Positive
Tetraniliprole	545.1	544	13.65	121	5	1	Positive
Tetraniliprole	545.1	375.3	13.65	121	5	28	Positive
Tetraniliprole	545.1	355.2	13.65	121	5	8	Positive
Tetraniliprole	545.1	272	13.65	121	5	40	Positive
Tetraniliprole	545.1	219	13.65	121	5	40	Positive
Tetraniliprole	545.1	185.2	13.65	121	5	40	Positive
Tetraniliprole	545.1	111.8	13.65	121	5	76	Positive
Triflumizol	346.1	73.1	17.3	91	2	17	Positive
Triflumizol	346.1	43.2	17.3	91	2	37	Positive
Triflumizole metabolite FM-6-1	295.1	214.9	9.4	93	5	25	Positive
Triflumizole metabolite FM-6-1	295.1	73.1	9.4	93	5	18	Positive
Triflumizole metabolite FM-6-1	295.1	55.2	9.4	93	5	22	Positive
Triflumuron	359	156	16.9	94	7	12	Positive
Triflumuron	359	139	16.9	94	7	32	Positive
Tritosulfuron	446	221	11.6	120	3	16	Positive
Tritosulfuron	446	195	11.6	120	3	20	Positive
Tritosulfuron	446	145	11.6	120	3	40	Positive

Table S3. List of pesticides, along with total retention time (TR) [min], precursor [*m/z*], product [*m/z*],, fragmentor, CAV [V] and ion transitions with collision energy - CE [eV], and polarity for method 1 based in QuEChERS method for GC-MS/MS.

Compound name	Precursor *m/z*	Product *m/z*	RT (min)	CE (V)
Acrinathrin	288.9	92.8	30.77	10

Acrinathrin	207.8	181.1	30.77	10
Acrinathrin	181	152	30.77	30
Benfluralin	292	264	11.73	5
Benfluralin	292	206	11.73	10
Bifenthrin	181.2	166.2	28.89	10
Bifenthrin	181.2	165.2	28.89	25
Chlorfenapyr	246.9	227	25.302	15
Chlorfenapyr	136.9	102	25.302	15
Cyflufenamid	188.1	88	25.28	35
Cyflufenamid	118.1	90	25.28	10
Cyflufenamid	118.1	89	25.28	25
Cyhalothrin (sum of isomers)	208	181	30.44	5
Cyhalothrin (sum of isomers)	197	141	30.44	10
Diflufenican	266	246.1	27.82	15
Diflufenican	266	238.1	27.82	15
Fipronil	366.8	212.8	21.80	25
Fipronil	350.8	254.8	21.80	15
Fipronil sulfone	384.8	256.8	24.81	20
Fipronil sulfone	382.8	254.9	24.81	20
Flonicamid	174	146	10.16	10
Flonicamid	174	69	10.16	40
Fludioxonil	248	182.1	24.08	10
Fludioxonil	248	154.1	24.08	20
Fluensulfone	226	206	10	20
Fluensulfone	188	96	10	35
Fluensulfone	121	94	10	10
Fluensulfone	119	92	10	10
Flufenacet	151	136.1	19.65	10
Flufenacet	151	95	19.65	30
Flumetralin	143	117	23.34	20
Flumetralin	143	107.1	23.34	20
Fluopyram	223	196	21.74	15
Fluopyram	173	145	21.74	15
Flurochloridone	311	174.1	19.95	15
Flurochloridone	187.1	159.1	19.95	10
Flutolanil	280.9	173	23.82	10
Flutolanil	173	145.1	23.82	15
Tau-fluvalinate	250	200	34.85	22
Tau-fluvalinate	250	55	34.85	18

Indoxacarb	202.9	134	35.99	15
Indoxacarb	202.9	106	35.99	25
Isopyrazam	359	282.1	30.99	23
Isopyrazam	359	159	30.99	21
Oxyfluorfen	252	196	24.72	20
Oxyfluorfen	252	146	24.72	30
Penthiopyrad	359	177	26.04	21
Penthiopyrad	359	152	26.04	17
Picoxystrobin	145	115.1	23.80	15
Picoxystrobin	145	102.1	23.80	25
Picolinafen	376	239.1	28.81	10
Picolinafen	376	238.1	28.81	20
Picolinafen	238.1	145.1	28.81	25
Pyridalyl	204	176.1	33.55	10
Pyridalyl	204	148.1	33.55	20
Pyridalyl	163.8	146.1	33.55	12
Tefluthrin	197	141.1	15.08	10
Tefluthrin	177.1	127.1	15.08	15
Trifloxystrobin	116	89	27.34	15
Trifloxystrobin	116	63	27.34	30
Triflumizole	206	186	22.33	10
Triflumizole	206	179	22.33	15
Trifluralin	305.9	264	11.64	5
Trifluralin	264	160.1	11.64	15

T**able S4.** List of pesticides, along with total retention time (TR) [min], precursor [*m*/*z*], product [*m*/*z*], fragmentor, CAV [V] and ion transitions with collision energy - CE [eV], and polarity for method 2 for LC-MS/MS.

**Compound name**	**Precursor *m*/*z***	**Product *m*/*z***	**RT (min)**	**Fragmentor (V)**	**CAV (V)**	**CE (V)**	**Polarity**
Flazasulfuron	408	181.7	18.8	121	5	16	Positive
Flazasulfuron	408	145.7	18.8	121	5	48	Positive
Flazasulfuron	408	138.7	18.8	121	5	44	Positive
Flazasulfuron	408	82.9	18.8	121	5	56	Positive
Flonikamid	230.1	203	12.1	110	4	15	Positive
Flonikamid	230.1	174	12.1	110	4	15	Positive
Fluazaindolizine	468	310.3	18.8	135	5	20	Positive
Fluazaindolizine	468	283.2	18.8	135	5	16	Positive
Fluazaindolizine	468	247	18.8	135	5	25	Positive
Fluazaindolizine	468	246	18.8	135	5	19	Positive
Fluazaindolizine	468	219.2	18.8	135	5	43	Positive
Fludioxonil metabolite CGA-192155	200.9	200.6	15.7	73	5	1	Negative
Fludioxonil metabolite CGA-192155	200.9	156.7	15.7	73	5	4	Negative

Fludioxonil metabolite CGA-192155	200.9	113	15.7	73	5	40	Negative
Fludioxonil metabolite CGA-192155	200.9	90.8	15.7	73	5	16	Negative
Fludioxonil metabolite CGA-192155	200.9	64.9	15.7	73	5	40	Negative
Fluometuron	231.1	186	18.2	93	5	18	Negative
Fluometuron	231.1	166	18.2	93	5	26	Negative
Fluometuron	231.1	146	18.2	93	5	26	Negative
Fluopyram benzamide (M25)	190.1	170	13.5	105	5	20	Positive
Fluopyram benzamide (M25)	190.1	150	13.5	105	5	20	Positive
Fluopyram benzamide (M25)	190.1	129.8	13.5	105	5	20	Positive
Fluopyram benzamide (M25)	190.1	101.9	13.5	105	5	36	Positive
Fluopyram benzamide (M25)	190.1	75	13.5	105	5	56	Positive
Isoxaflutole	360	251	19.1	95	4	20	Positive
Isoxaflutole	360	220	19.1	95	4	35	Positive
Isoxaflutole	360	144	19.1	95	4	60	Positive
Isoxaflutole diketonitrile	377	360	16.2	62	5	8	Positive
Isoxaflutole diketonitrile	377	250.9	16.2	62	5	25	Positive
Isoxaflutole diketonitrile	360	69.1	16.2	135	5	9	Positive
Mefentrifluconazole	398.1	182	23	137	5	32	Positive
Mefentrifluconazole	398.1	167.1	23	137	5	64	Positive
Mefentrifluconazole	398.1	133.1	23	137	5	92	Positive
Mefentrifluconazole	398.1	70.2	23	137	5	24	Positive
Penoxsulam	484	483.1	17.8	169	5	1	Positive
Penoxsulam	484	443.2	17.8	169	5	24	Positive
Penoxsulam	484	326.1	17.8	169	5	40	Positive
Penoxsulam	484	194.7	17.8	169	5	28	Positive
Penoxsulam	484	166	17.8	169	5	40	Positive
Penoxsulam	484	164.1	17.8	169	5	40	Positive
Penoxsulam	484	123.8	17.8	169	5	60	Positive
Prosulfuron	420.1	167	19.4	120	3	10	Positive
Prosulfuron	420.1	141.1	19.4	120	3	15	Positive
Pyroxsulam	435.1	195.1	17.2	155	3	24	Positive
Pyroxsulam	435.1	165.7	17.2	155	3	40	Positive
Pyroxsulam	435.1	124.1	17.2	155	3	48	Positive
Tetraniliprole	545	544	18.9	121	5	1	Positive
Tetraniliprole	545	375.3	18.9	121	5	28	Positive
Tetraniliprole	545	355.2	18.9	121	5	8	Positive
Tetraniliprole	545	272	18.9	121	5	40	Positive
Tetraniliprole	545	219	18.9	121	5	40	Positive
Tetraniliprole	545	185.2	18.9	121	5	40	Positive
Tetraniliprole	545	111.8	18.9	121	5	76	Positive
TFNA (Trifluoroacetic acid derivative)	190	146	5.4	65	4	13	Negative
TFNA (Trifluoroacetic acid derivative)	190	126	5.4	65	4	25	Negative
TFNA (Trifluoroacetic acid derivative)	190	69	5.4	65	4	33	Negative

TFNG (Trifluoroacetic acid glucoside)	247	183	9.5	86	4	9	Negative
TFNG (Trifluoroacetic acid glucoside)	247	163	9.5	86	4	13	Negative
TFNG (Trifluoroacetic acid glucoside)	247	146	9.5	86	4	17	Negative
Triflumizole metabolite FM-6-1	295.1	214.9	18.1	93	5	25	Positive
Triflumizole metabolite FM-6-1	295.1	73.1	18.1	93	5	18	Positive
Triflumizole metabolite FM-6-1	295.1	55.2	18.1	93	5	22	Positive
Triflusulfuron metabolite IN-M72222	210	167.9	12.5	135	5	40	Positive
Triflusulfuron metabolite IN-M72222	210	125.2	12.5	135	5	40	Positive
Triflusulfuron metabolite IN-M72222	210	110.1	12.5	135	5	40	Positive
Triflusulfuron metabolite IN-M72222	210	86	12.5	135	5	40	Positive
Triflusulfuron metabolite IN-M72222	210	83.1	12.5	135	5	40	Positive
Tritosulfuron metabolite AMTT	195	194.6	13	113	5	1	Positive
Tritosulfuron metabolite AMTT	195	175.2	13	113	5	40	Positive
Tritosulfuron metabolite AMTT	195	83	13	113	5	20	Positive

Table S5. List of pesticides, along with total retention time (TR) [min], precursor [*m/z*], product [*m/z*], fragmentor, CAV [V] and ion transitions with collision energy - CE [eV], and polarity for method 1 based in QuPPe method for LC-MS/MS.

Compound Name	Precursor *m/z*	Product *m/z*	RT (min)	Fragmentor	CAV (V)	CE (V)	Polarity
Difluoroacetic acid (DFA)	95	95	4	28	3	5	Negative
Difluoroacetic acid (DFA)	95	51	4	28	3	9	Negative
Trifluoroacetic acid (TFA)	113.1	113.1	5	46	3	1	Negative
Trifluoroacetic acid (TFA)	113.1	69.2	5	46	3	9	Negative

Table S6. LogP values of the pesticides and pesticide metabolites included in the study.

Compound	logP
Acrinathrin	6.8
Beflubutamid	4.6
Benfluralin	5.3
Benzobicyclon	4.4
Bifenthrin	6.0
Chlorfenapyr	4.5
Chlorfluazuron	6.7
Cyflufenamid	5.0
Cyflumetofen	5.5

Cyhalothrin (sum of isomers)	6.1
Difluoroacetic acid (DFA)	0.6
Diflubenzuron	3.9
Diflufenican	4.6
Epoxiconazole	3.2
Fipronil	4.5
Fipronil sulfone	4.7
Flazasulfuron	2.0
Flonicamid	0.8
Florasulam	1.6
Fluazaindolizine	4.6
Fluazinam	6.0
Flubendiamide	5.3
Fludioxonil	2.6
Fludioxonil metabolite CGA 192155	–
Fluensulfone	2.5
Flufenacet	3.6
Flufenoxuron	6.3
Flumetralin	5.2
Fluometuron	2.4
Fluopicolide	4.4
Fluopyram	4.5
Fluopyram benzamide (M25)	4.5
Fluquinconazole	–
Flurochloridone	3.4
Flupyradifurone	2.3
Flutianil	5.6
Flutolanil	3.7
Fluxapyroxad	3.7
Hexaflumuron	5.7
Hydramethylnon	6.4
Indoxacarb	4.8
Isopyrazam	4.0
Isoxaflutole	2.4
Isoxaflutole diketonitrile	2.0
Lufenuron	6.4
Mefentrifluconazole	3.4
Metaflumizone	6.2
Norflurazon	2.3
Novaluron	5.8
Oxathiapiprolin	4.4
Oxyfluorfen	4.7
Penflufen	4.4
Penoxsulam	2.9

Penthiopyrad	4.0
Picolinafen	4.9
Picoxystrobin	3.6
Prosulfuron	3.4
Pydiflumetofen	4.7
Pyridalyl	7.1
Pyroxsulam	1.7
Sulfoxaflor	2.2
Tau-fluvalinate	7.7
Tefluthrin	5.4
Tetraconazole	4.4
Tetraniliprole	3.6
TFNA (4-(trifluoromethyl)nicotinic acid)	1.3
TFNG (N-(4-trifluoromethylnicotinoyl)glycine)	0.6
Trifloxystrobin	4.9
Trifluoroacetic acid (TFA)	0.9
Triflumizole	4.0
Triflumizole metabolite FM-6-1	3.3
Trifluralin	5.3
Triflumuron	5.6
Triflusulfuron metabolite IN-M72222	–
Tritosulfuron	3.1
Tritosulfuron metabolite AMTT	2.0

Figure S1. Representative MRM chromatograms of DFA: (A) reagent blank; (B) blank pear-matrix extract; and (C) pear matrix-matched calibration standard at the validated LOQ of 0.020 mg kg^{-1}.

(A) Reagent blank

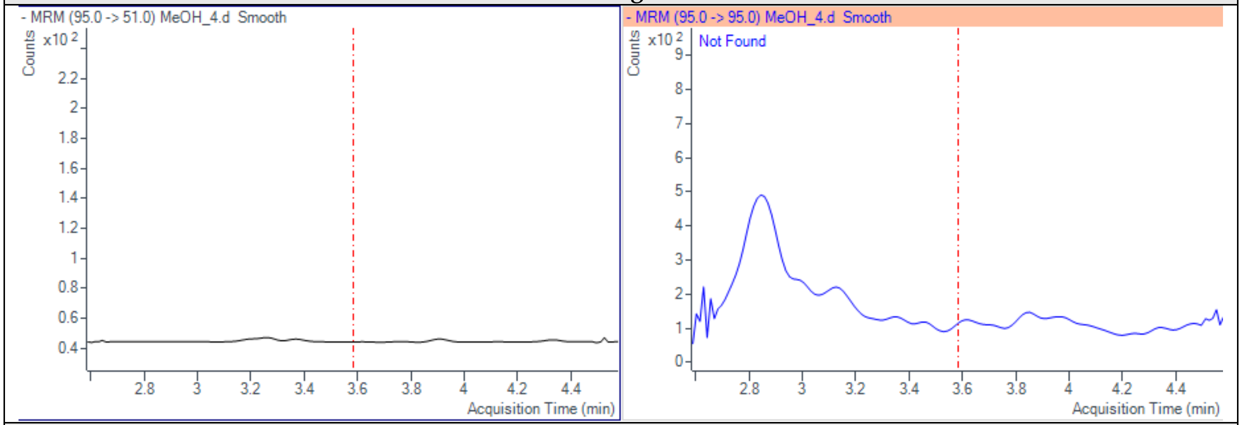

(B) Blank pear matrix

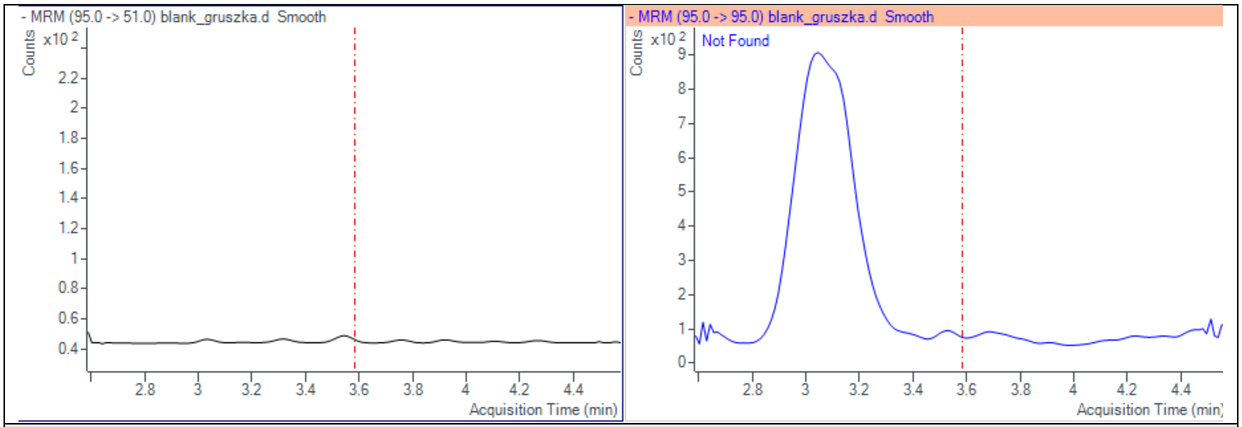

(C) Matrix-matched standard at the validated LOQ 0.020 mg kg^{-1}

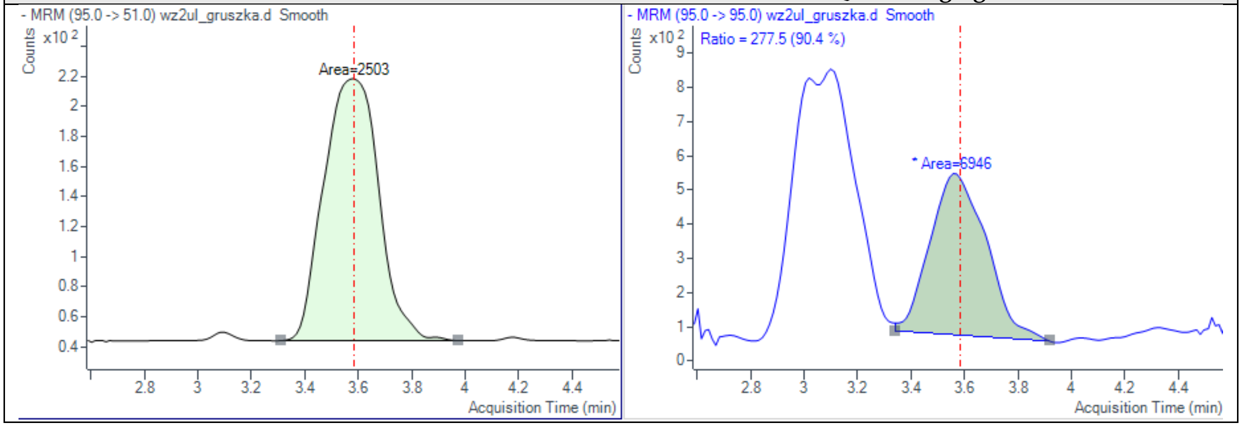

Figure S2. Representative MRM chromatograms of TFA: (A) reagent blank; (B) blank pear-matrix extract; and (C) pear matrix-matched calibration standard at the validated LOQ of 0.050 mg kg^{-1}.

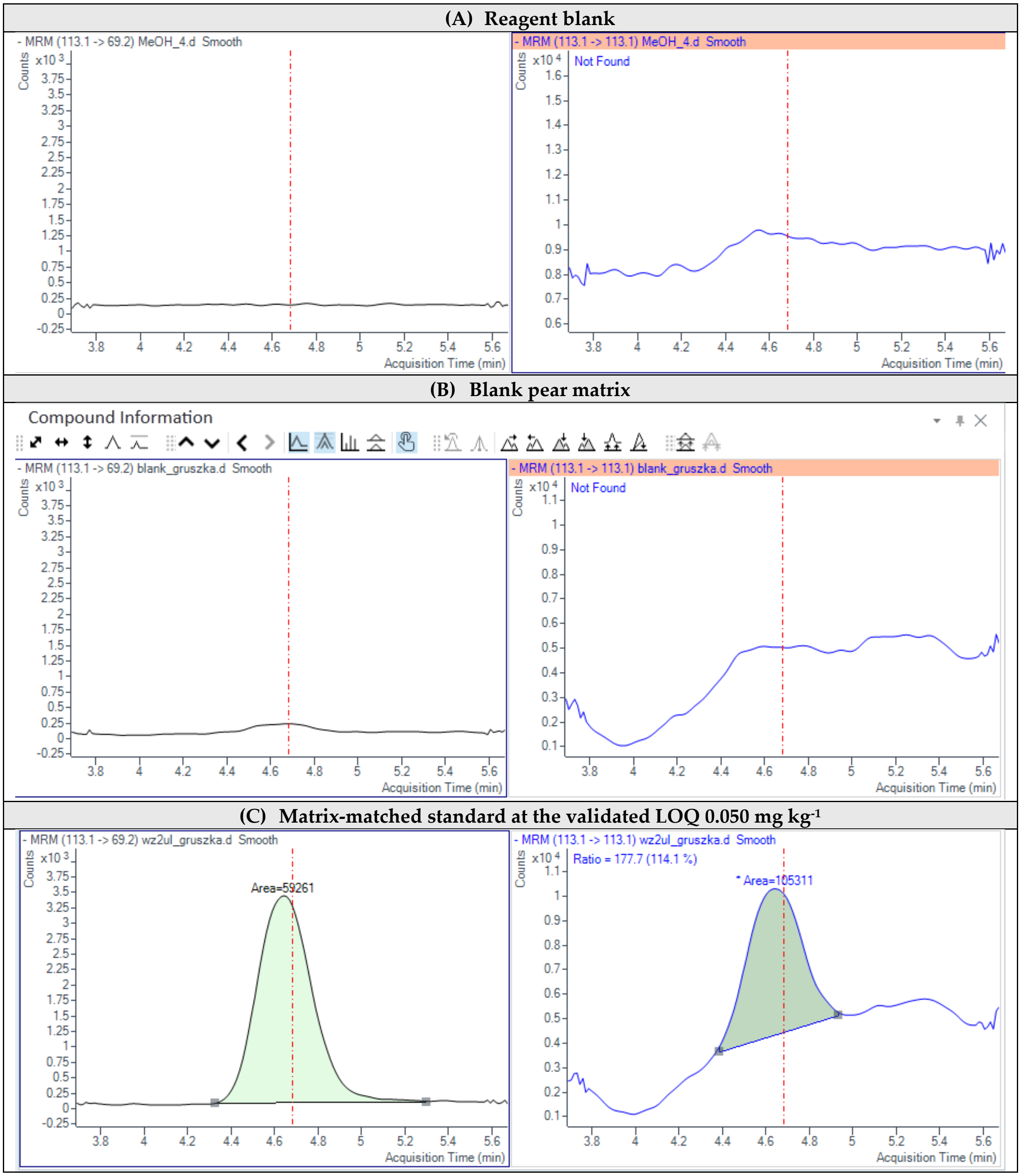

Supplement: Supplementary file 1 [file foods-15-03050-s001.zip › foods-4482069-supplementary.pdf]
